# WFDC3 inhibits tumor metastasis by promoting the ERβ-mediated transcriptional repression of TGFBR1 in colorectal cancer

**DOI:** 10.1038/s41419-023-05956-0

**Published:** 2023-07-13

**Authors:** Tianqi Liu, Min Zhao, Lin Peng, Jiangbo Chen, Pu Xing, Pin Gao, Lei Chen, Xiaowen Qiao, Zaozao Wang, Jiabo Di, Hong Qu, Beihai Jiang, Xiangqian Su

**Affiliations:** 1 Key Laboratory of Carcinogenesis and Translational Research (Ministry of Education), Department of Gastrointestinal Surgery IV, Peking University Cancer Hospital & Institute, 100142 Beijing, China; 2 School of Science, Technology and Engineering, University of the Sunshine Coast, Maroochydore DC, Sunshine Coast, QLD 4556 Australia; 3 Center for Bioinformatics, State Key Laboratory of Protein and Plant Gene Research, College of Life Sciences, Peking University, 100871 Beijing, People’s Republic of China

**Keywords:** Metastasis, Gastrointestinal cancer, Tumour-suppressor proteins

## Abstract

Estrogen plays a protective role in colorectal cancer (CRC) and primarily functions through estrogen receptor β (ERβ). However, clinical strategies for CRC therapy associated with ERβ are still under investigation. Our discoveries identified WFDC3 as a tumor suppressor that facilitates estrogen-induced inhibition of metastasis through the ERβ/TGFBR1 signaling axis. WFDC3 interacts with ERβ and increases its protein stability by inhibiting its proteasome-dependent degradation. WFDC3 represses TGFBR1 expression through ERβ-mediated transcription. Blocking TGFβ signaling with galunisertib, a drug used in clinical trials that targets TGFBR1, impaired the migration of CRC cells induced by WFDC3 depletion. Moreover, there was clinical significance to WFDC3 in CRC, as CRC patients with high WFDC3 expression in tumor cells had favorable prognoses. Therefore, this work suggests that WFDC3 could be an indicator for therapies targeting the estrogen/ERβ pathway in CRC patients.

## Introduction

Colorectal cancer (CRC) is the third most common cancer worldwide with a high risk of metastasis [1]. The CRC morbidity and mortality rates are higher in males than in females, possibly because of the protective effect of female sex steroid hormones [2]. Hormone replacement therapy in postmenopausal women was correlated with a lower risk for developing CRC [3, 4]. A better understanding of the underlying mechanisms of the estrogen-related process in CRC is necessary to develop new therapies and improve outcomes in patients.

Estrogens exert their biological functions through the estrogen receptors (ERs), including ERα and ERβ. ERα and ERβ are antagonistic to each other and can regulate tumor progression in opposite ways [5]. Unlike ERα, which is primarily expressed in breast cancer tissues and promotes estrogen-related tumor metastasis, high expression of ERβ was confirmed in CRC tissues and was found to be associated with the suppression of CRC progression [6]. Loss of ERβ expression in CRC tissues indicates worse tumor differentiation and more advanced cancer stage in CRC patients [6, 7]. ERβ-mediated autophagy facilitates CRC cell growth inhibition by inducing cyclin D1 degradation [8]. Moreover, as a transcription factor, ERβ can both upregulate and downregulate the transcriptional levels of target genes and play essential roles in tumor progression [9–11]. Because of these various roles of ERβ in tumor progression, the underlying mechanism of the ERβ signaling pathway in CRC requires further investigation.

About 25% of CRC patients develop liver metastasis that results in a high risk of cancer-related death [12]. In the early steps of metastasis, the EMT plays a crucial role in the cancer invasion and metastasis processes [13]. Numerous signaling pathways are involved in regulating the EMT process and tumor metastasis, such as the PI3K/AKT, Wnt/β-catenin, Notch, and TGFβ/SMAD signaling pathways. The TGFβ pathway is activated when TGFβ binds to its corresponding receptors, TGFBR1 and TGFBR2. Blockade of TGFβ signaling by galunisertib, which targets the TGFBR1, improved response rates to neoadjuvant chemoradiotherapy in patients with advanced rectal cancer [14]. Previous studies have revealed the crosstalk between the ERβ and TGFβ pathways in cancer progression [15–17]. Jordan et al. found that ERβ-mediated induction of cystatins can lead to the suppression of TGFβ signaling and the inhibiton of breast cancer metastasis [16]. Moreover, ERβ was required for the estrogen-inhibited TGFBR1 expression and metabolism of osteoblasts [17]. However, the relationship between the ERβ and TGFβ signaling pathways in CRC metastasis remains uncharacterized.

The whey acidic protein (WAP) four-disulfide core domain (WFDC) family of genes have multiple active functions, including as anti-microbials, anti-HIV agents, and immune regulators [18]. These genes are also involved in the progression of cancers [19]. Interestingly, the roles of WFDC family members WFDC4, WFDC14, and WFDC2 are different in various cancers [20–23]. High WFDC4 (SLPI) expression levels indicate shorter overall survival (OS) in CRC patients with liver metastases [20]. WFDC14 (Elafin) could bind to EGFR and trigger hepatocellular carcinoma (HCC) metastasis via activation of EGFR/AKT signaling [21]. WFDC2 can interact with annexin II, promoting ovarian cancer cell metastasis via the MAPK and FOCAL adhesion pathways [22]. However, WFDC2 inhibits prostate cancer metastasis by suppressing EGFR signaling [23]. Because of the controversial and contradicting effects of WFDC family members, the function of WFDC3 in different cancer types needs to be elucidated.

WFDC3 may participate in lipopolysaccharide (LPS)-induced inflammation [24] and the occurrence of Systemic Lupus Erythematosus-related disease [25]. Previous studies suggest that WFDC3 was one of the most downregulated genes in the ventral prostate of ERβ-/- (ERβ knockout) mice, indicating the potential role of WFDC3 in the ERβ pathway [26]. Whether WFDC3 can influence the effects of estrogen/ERβ signaling in CRC progression still remains to be investigated.

In this study, WFDC3 was first identified as a regulator of the estrogen/ERβ pathway via mediating ERβ ubiquitination and stability, which subsequently repressed TGFBR1 transcription and CRC metastasis. Moreover, CRC patients with high WFDC3 expression had more favorable prognoses. Taken together, these findings suggest that WFDC3 could be an indicator for therapies targeting the estrogen/ERβ pathway in CRC patients.

## Materials and methods

### Animal models and treatment

The experiments were performed according to experimental animal management ordinance and approved by the Ethics Committee of the Peking University Cancer Hospital & Institute (No. 2021KT134).

Female nude mice (BALB/c, 6-weeks old) were obtained from the Hua-Fu-Kang Corporation (Beijing, China). To investigate the effects of WFDC3 on tumor growth in vivo, RKO cells stably expressing WFDC3 (LV-WFDC3) or control cells (LV-vector) were injected subcutaneously into nude mice. Tumor growth was measured every 3 days using calipers. Tumor volume was calculated by the following formula: 0.5 × *L* × *W*^2^. At 25 days after tumor inoculation, all mice were sacrificed to collect tumor xenografts and tumor xenografts were weighted.

To investigate the effects of WFDC3 and estrogen on metastasis in vivo. Mice were randomly subcutaneously implanted with 0.72 mg/60-day release 17β-estradiol (E2) pellet (SE121, Innovative Research of America, Sarasota, FL, USA) or a corresponding placebo pellet. After 5 days, the liver metastasis model was generated by injecting 5 × 10^6^ luciferized LoVo cells stably expressing WFDC3 (LV-WFDC3) or control cells (LV-vector) into the spleens of mice. The mice were divided into four groups (5 mice/group): (1) LV-vector + placebo; (2) LV-WFDC3 + placebo; (3) LV-vector + E2; (4) LV-WFDC3 + E2.

For imaging the tumor metastasis, mice were anesthetized with isoflurane and injected with 100 μL luciferin substrate before in vivo imaging. Liver metastasis was monitored by in vivo bioluminescence imaging using an IVIS system (PerkinElmer, Hopkinton), and average radiance was quantified with Living Image software.

Mice were sacrificed four weeks after injection, and the metastatic livers were then harvested, fixed with 4% PFA, and the number of metastatic nodules was counted. The tissues were then embedded in paraffin for hematoxylin and eosin (HE) and immunohistochemistry (IHC) staining. The investigators were blinded to the group allocation during the experiment.

### Co-immunoprecipitation (Co-IP) assays

For Co-IP analysis of Flag-WFDC3 and Myc-ERβ, total protein (500 µg) from the transfected cells was incubated with Protein A Sepharose beads (GE Healthcare, US) for 2 h at 4 °C. Then, immunoprecipitation was performed with 1 µg of c-Myc antibody or Flag antibody at 4 °C overnight. The beads were washed thoroughly with ice-cold lysis buffer, followed by western blot analysis.

### Luciferase assay

To examine the transcriptional regulation of ERβ on TGFBR1, the potential binding sites of ERβ in the TGFBR1 promoter (from −1000 bp to 0 bp) were predicted using the JASPAR database. The fragments of the TGFBR1 promoter ( − 1000 bp to 0 bp) and the deletion mutant containing the corresponding ERβ-binding sites deletion (mut1: −376 bp to −361 bp, and mut2: −313 bp to −298 bp) were inserted into the pGL3-basic plasmid (Promega). CRC cells were plated in 24-well plates and transfected with the luciferase reporter plasmid. Luciferase reporter assays were performed using a Dual-Luciferase Reporter system according to the manufacturer’s instructions (Promega). Firefly luciferase activities were normalized to the Renilla luciferase activities.

### Patients and tissue samples

A total of 173 CRC samples were obtained from patients that received surgical resection in the Department of Gastrointestinal Surgery IV, Peking University Cancer Hospital & Institute from 2009 to 2012. Patients did not receive preoperative chemotherapy or chemoradiotherapy. This research was performed in accordance with the Declaration of Helsinki and approved by the Ethics Committee of Peking University Cancer Hospital & Institute (No. 2021KT134) with written informed consent obtain from all patients. CRC samples were collected for analysis by IHC, western blot and qRT-PCR analysis.

### Immunohistochemistry and staining quantification

IHC staining of paraffin-embedded human CRC samples or mice liver sections were performed following standard protocols [22]. For human CRC samples, the rabbit anti-WFDC3 (1:50, 24917-1-AP, Proteintech) was used as the primary antibody. The staining level of WFDC3 was quantified according to the staining intensity (0, no staining; 1, weak staining; 2, strong staining) and the percentage of positively stained cells (0, 0% stained; 1, 1–25% stained; 2, 26–50% stained; 3, 51–75% stained; 4, 76–100% stained). The immunohistochemical staining was evaluated by two experienced pathologists in a double-blinded manner. The intensity and percentage score of each sample were multiplied to obtain a total IHC score of 0–8. We classified the WFDC3 staining into two categories: negative expression (score of <1) and positive expression (score of ≥1).

For mice liver sections, N-cadherin mAb (1:125, 13116S, CST, USA) and E-cadherin mAb (1:400, 3195, CST) were used as primary antibodies.

### Statistical analyses

To identify the hallmark effect gene sets associated with WFDC3 mRNA expression in the Cancer Genome Atlas (TCGA, https://tcga-ddata.nci.nih.gov/docs/publications/tcga/), gene set enrichment analysis (GSEA) was performed using the GSEA software and hallmark signatures.

Statistical analysis was performed using SPSS software (version 24.0) and GraphPad Prism (version 9.0). Data are shown as mean ± standard deviation (SD). Student’s *t*-test were conducted to compare two groups. Pearson *χ*^2^ tests were used to analyze the associations of WFDC3 expression and clinicopathologic features. Kaplan–Meier curve analysis was performed to create survival curves for overall survival (OS) and disease-free survival (DFS), which were compared with the log-rank test. Cox proportional hazard regression models were applied for univariate and multivariate survival analyses. A *P*-value < 0.05 was considered statistically significant. All experiments were performed three times.

## Results

### WFDC3 increased the inhibitory effect of estrogen on migration and invasion of CRC cells

**Fig. 1:**
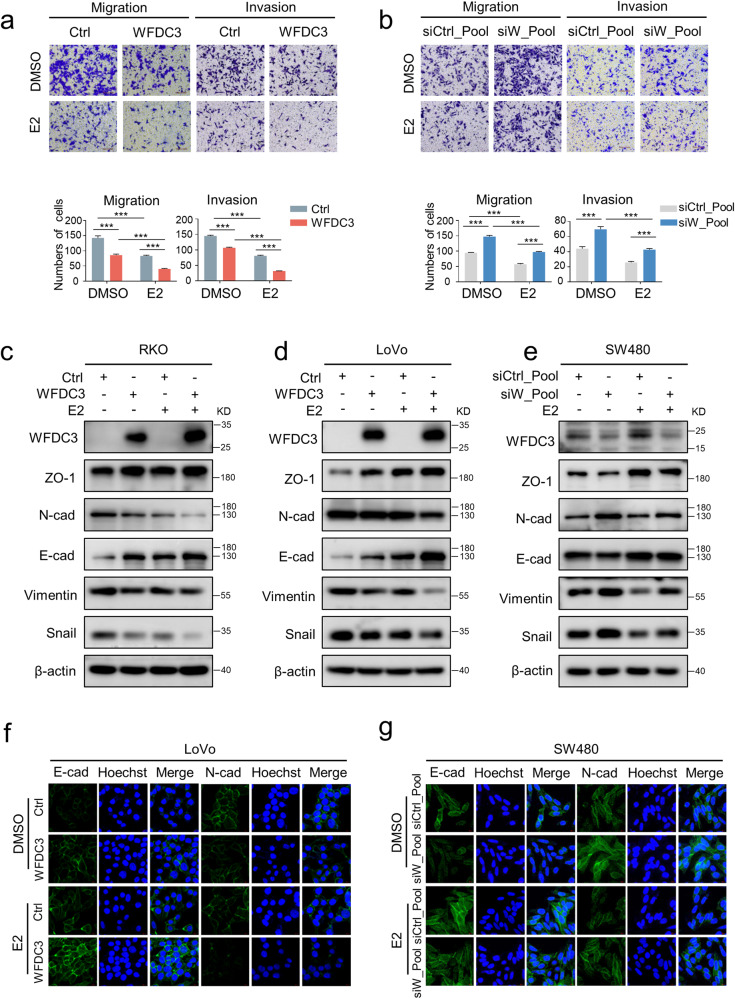
WFDC3 enhances the inhibitory effects of estrogen on migration and EMT. **a** and **b** Transwell migration and Matrigel invasion assays performed in LoVo cells transfected with WFDC3 plasmid (**a**) or SW480 cells transfected with pooled WFDC3 siRNA (**b**) and treated with estrogen or DMSO control. The bar graph indicates the number of migrated cells. **c–e** Western blot analysis of ZO-1, N-cadherin, E-cadherin, Vimentin, and Snail protein levels in RKO cells (**c**), LoVo cells (**d**) transfected with WFDC3 plasmid, and in SW480 cells treated with pooled WFDC3 siRNA (**e**). **f** and **g** The epithelial marker E-cadherin and mesenchymal marker N-cadherin were detected by immunofluorescence staining in LoVo cells (**f**) and SW480 (**g**) cells treated as in **a** and **b**. Nuclei were counterstained with Hoechest 33342 (blue). Data are expressed as mean ± SD of at least three independent experiments. Statistics were performed using unpaired Student’s *t*-test, ***P < 0.001.

A previous study found that estrogen could inhibit the metastatic potential of CRC [27], which was confirmed by our experiments in which 17β-estradiol (E2) treatment decreased the migration and invasion of CRC cells (Fig. 1a, b). To investigate the involvement of WFDC3 in CRC cell functions with or without E2 treatment, we first examined the effects of modulating WFDC3 levels in four CRC cell lines. RKO and LoVo cells with relatively low WFDC3 expression were transfected with a WFDC3 expression plasmid (Fig. S1a). HCT116 and SW480 cells with relatively high WFDC3 expression were transfected with a mixture of four individual siRNA (siRNAs) to silence WFDC3 (Fig. S1b). We then examined the cellular phenotypes related to proliferation, migration, and invasion. Overexpression or knockdown of WFDC3 had no remarkable effects on cell proliferation, colony formation in CRC cells (Fig. S1c–f), or the growth of CRC xenografts in nude mice (Fig. S1g–i). However, an inhibition of cell migration and invasion by WFDC3 overexpression were validated by transwell migration and wound healing assays (Fig. 1a and Fig. S2a, b). Conversely, knocking down WFDC3 significantly increased CRC cell migration and invasion (Fig. 1b and Fig. S2c, d).

We next examined whether WFDC3 was involved in the estrogen-induced inhibition of migration. Interestingly, we found that overexpressing WFDC3 promoted the E2 -induced inhibition of migration in CRC cells (Fig. 1a and Fig. S2a, b). In contrast, silencing WFDC3 protected cancer cells from E2 (Fig. 1b and Fig. S2c, d). Taken together, these results indicate that WFDC3 can inhibit the migration of CRC cells and enhance the effects of estrogen-mediated inhibition of CRC migration.

### WFDC3 suppressed EMT and promoted estrogen-mediated EMT inhibition

EMT is a critical step for the initiation of the metastatic cascade because it facilitates increased cancer cell motility and acquisition of invasive features [13]. Given that estrogen treatment affects the EMT process in several cancer types, including breast cancer [28], ovarian cancer [29], and lung cancer [30], we therefore reasoned that estrogen may also participate the EMT process in CRC. Thus, the effect of estrogen in the EMT process of CRC cells was investigated by both western blot and confocal immunofluorescence analyses. E2 drastically enhanced the levels of epithelial markers (E-cadherin, ZO-1), and decreased the levels of mesenchymal markers (N-cadherin, Vimentin, and Snail), suggesting that stimulation with estrogen inhibited the EMT process in CRC cells (Fig. 1c–g).

We further examined the effects of WFDC3 expression on the expression of EMT-related markers with or without E2 treatment. Overexpressing WFDC3 in RKO and LoVo cells led to a significant increase in E-cadherin, ZO-1 protein levels, as well as a decrease in N-cadherin, Vimentin, and Snail protein levels (Fig. 1c, d and Fig. S3a, b), suggesting decreased EMT process. Moreover, WFDC3 overexpression could enhance the E2 effects in EMT inhibition (Fig. 1c, d and Fig. S3a, b). Conversely, silencing WFDC3 in SW480 cells decreased the levels of E-cadherin, ZO-1, and increased expression of N-cadherin, Vimentin, and Snail (Fig. 1e and Fig. S3c). Additionally, E2-induced inhibition in EMT could be reversed by WFDC3 depletion (Fig. 1e and Fig. S3c). Confocal immunofluorescence of the WFDC3 overexpressing (Fig. 1f) or knockdown cells also confirmed these trends (Fig. 1g). These data suggest that WFDC3 can suppress EMT and enhance estrogen-mediated inhibition of EMT in CRC cells.

### WFDC3 inhibits metastasis and promotes estrogen-induced inhibition of metastasis in vivo

**Fig. 2:**
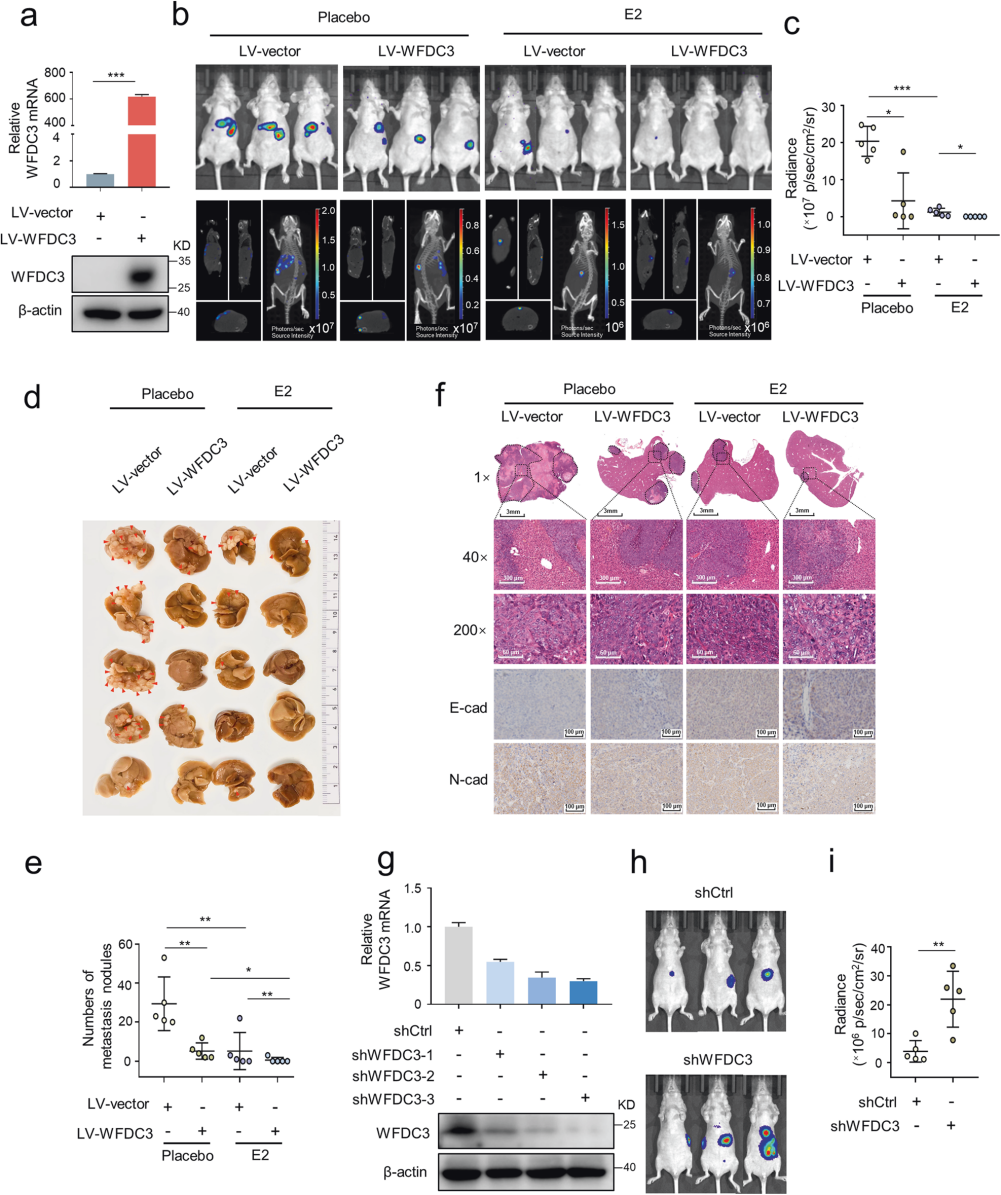
WFDC3 promotes estrogen-induced inhibition of metastasis in vivo. **a** qRT-PCR and western blot analyses verified the overexpression efficiency of WFDC3 in LoVo cells. **b** Two-dimensional (2D) and three-dimensional (3D) bioluminescence imaging of representative mice from each treatment group three weeks after implantation. **c** Quantification of 2D bioluminescence imaging. **d** Photographs of metastatic nodules in the liver of mice from each group. Arrows indicate metastatic nodules on liver surface. **e** Quantification of liver metastasis nodules. **f** Representative H&E staining images of liver metastatic lesions (magnification at 1×, 40×, and 200×) and IHC staining for E-cadherin and N-cadherin in the liver sections for each group. Data are expressed as mean ± SD (*n* = 5). **g** qRT-PCR and western blot analyses confirmed the knockdown efficiency of WFDC3 in HCT116 cells. shWFDC3-3 was used for subsequent in vivo analysis. **h** Two-dimensional (2D) bioluminescence imaging of representative mice of each group three weeks after implantation. **i** Quantification of 2D bioluminescence imaging. Statistics were performed using unpaired Student’s *t*-test, *P < 0.05, **P < 0.01, ***P < 0.001.

According to the role of WFDC3 in estrogen-mediated inhibition of CRC cell migration, we next sought to determine whether WFDC3 can affect E2 treatment in vivo. To this aim, we established a LV-WFDC3 LoVo cell model stably overexpressing WFDC3 (Fig. 2a). Similar to the in vitro results, E2 treatment delayed tumor metastasis compared with placebo treatment (Fig. 2b, c). The results also showed that mice injected with LV-WFDC3 cells had less metastatic potential compared with the LV-vector group, which were verified by decreased luminescence of the mouse liver (Fig. 2b, c). Accordingly, the number of macro- or micro-metastases nodules in the mouse livers was significantly decreased in the LV-WFDC3 group compared with the LV-vector group (Fig. 2d–f). Moreover, consistent with the in vitro results, WFDC3 enhanced the E2-mediated inhibition of CRC metastasis in vivo (Fig. 2b–f). These results suggest that WFDC3 can inhibit the metastatic potential of CRC cells in vivo and promote the inhibitory effects of estrogen on metastatic capacity.

We next examined if WFDC3 promoted the estrogen-mediated inhibition of EMT in vivo. Levels of E-cadherin and N-cadherin expression in metastatic liver tissues were measured by IHC. Compared with the LV-vector group, E-cadherin staining was much stronger in the LV-WFDC3 group, while N-cadherin staining was significantly reduced in the LV-WFDC3 group (Fig. 2f). These findings indicate that EMT was suppressed following WFDC3 overexpression. Furthermore, consistent with the in vitro findings, WFDC3 promoted estrogen-mediated EMT inhibition in vivo (Fig. 2f). Additionally, we established an shWFDC3 HCT116 cell model with stable knockdown of WFDC3 (Fig. 2g). The results showed that mice injected with shWFDC3 cells had more liver metastases than the shCtrl group (Fig. 2h, i).

### WFDC3-induced ERβ expression is required for estrogen-mediated inhibition of CRC cell migration and invasion

**Fig. 3:**
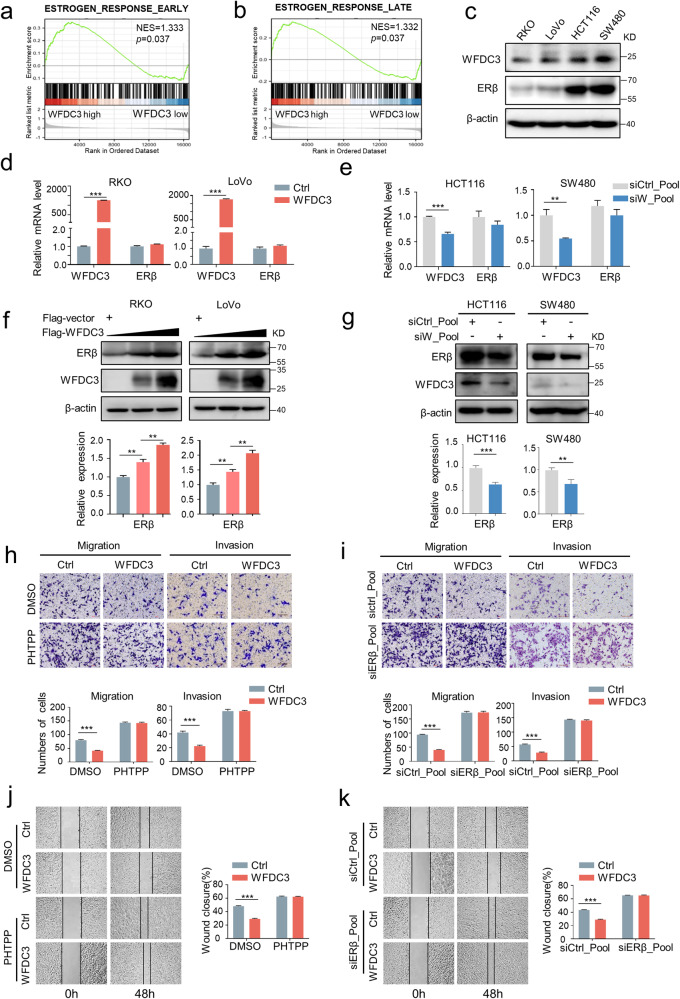
The effects of WFDC3 in estrogen-induced inhibition of metastasis are dependent on ERβ levels. **a** and **b** Early estrogen response (**a**) and late estrogen response (**b**) signaling was enriched in the WFDC3-high group in the hallmark gene sets database according to gene set enrichment analysis (GSEA). **c** Western blot analysis of WFDC3 and ERβ expression in CRC cell lines. **d** and **e** WFDC3 overexpression (**d**) or depletion (**e**) exerts no significant effect on ERβ mRNA expression levels. **f** WFDC3 overexpression increased the ERβ protein level in a plasmid concentration-dependent manner in RKO and LoVo cells. **g** WFDC3 depletion downregulates the protein levels of ERβ in HCT116 and SW480 cells. **h** and **i** Transwell migration and Matrigel invasion assays of the WFDC3-overexpressing LoVo cells treated with PHTPP (**h**) or a siERβ pool (**i**) in the presence of estrogen. **j** and **k** Rescue effects of treatment with estrogen combined with PHTPP (**j**) or a siERβ pool co-transfection (**k**) on WFDC3 overexpression-mediated inhibition of cell migration in LoVo cells determined by wound healing assays. The bar graph indicates the number of migrated cells and percentage of wound closures. Data are expressed as mean ± SD of at least three independent experiments. Statistics were performed using unpaired Student’s *t*-test, ***P < 0.001.

To determine the possible molecular mechanism underlying how WFDC3 enhances metastatic inhibition induced by estrogen, we performed a gene set enrichment analysis (GSEA) to annotate the hallmark effector gene sets associated with WFDC3 levels in the Cancer Genome Atlas (TCGA) datasets, which contains colon and rectal adenocarcinoma data. The results revealed that both the early and late estrogen response pathways were positively correlated with high WFDC3 levels (Fig. 3a, b)

In CRC, estrogen primarily activates ERβ [6]. Thus, we investigated whether WFDC3-induced inhibition of cell migration is dependent on ERβ levels. First, we evaluated the possible correlation between WFDC3 and ERβ expression levels in CRC. Four cell lines were examined for mRNA and protein expression of WFDC3 and ERβ by qRT-PCR and western blots, respectively. The mRNA and protein levels of WFDC3 and ERβ were positively correlated in RKO, LoVo, and SW480 cells, but not in HCT116 cells. (Fig. 3c and Fig. S4a, b). However, as compared to robust ERα expression in MCF-7 breast cancer cells, ERα expression in CRC cells was undetectable (Fig. S4c).

The association between WFDC3 and ERβ led us to examine the effects of WFDC3 on ERβ expression. Overexpression and knockdown of WFDC3 showed few effects on ERβ mRNA levels (Fig. 3d, e). However, WFDC3 overexpression significantly increased ERβ expression in a plasmid concentration-dependent manner (Fig. 3f), whereas knockdown of WFDC3 decreased ERβ expression (Fig. 3g). These results indicate that WFDC3 can regulate ERβ expression at the post-transcriptional level rather than transcriptional level. However, overexpressing or silencing ERβ had no obvious influence on WFDC3 mRNA or protein expression levels in CRC cells (Fig. S4d–g).

Given that WFDC3 can regulate ERβ expression, we next sought to investigate whether WFDC3-mediated ERβ expression was required for the estrogen-induced inhibition of migration in CRC cells. As expected, overexpression of ERβ decreased the migration of LoVo cells (Fig. S5a, b). Inhibiting ERβ with its antagonist (PHTPP) reversed this effect (Fig. S5a, b). Following E2 treatment, WFDC3 overexpression significantly inhibited the migration of CRC cells. However, PHTPP and ERβ depletion rescued WFDC3-impaired CRC cell migration with E2 treatment (Fig. 3h-k), indicating WFDC3-mediated inhibition in migration is dependent on ERβ signaling activity. These results revealed a functional significance of WFDC3-induced ERβ expression in estrogen-mediated migration inhibition in CRC.

### WFDC3 directly interacts with ERβ and inhibits its degradation via the ubiquitin-proteasome pathway

**Fig. 4:**
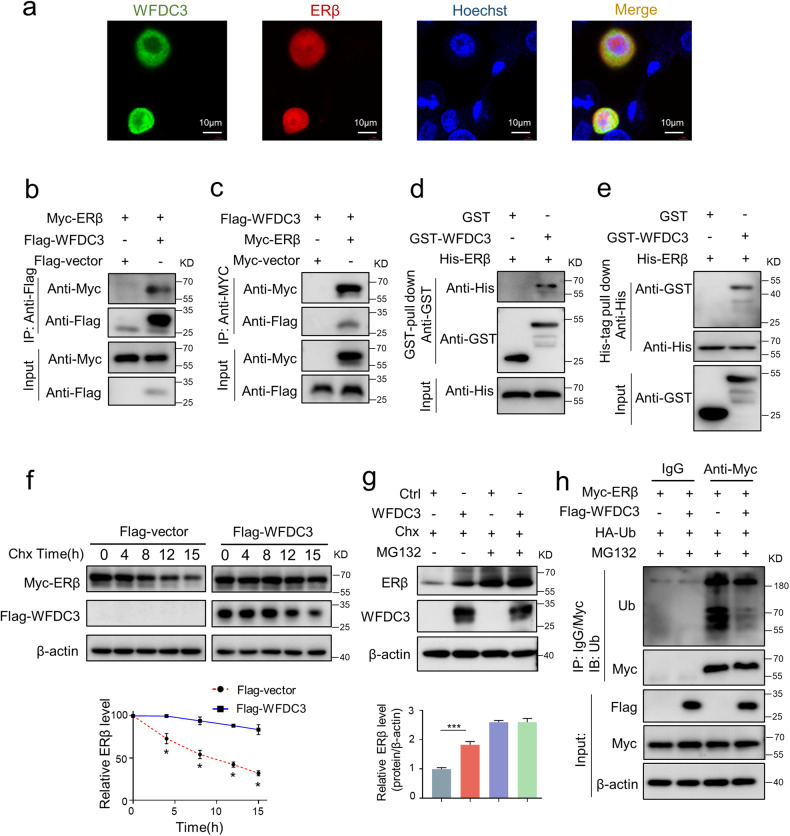
WFDC3 interacts with and stabilizes ERβ protein by inhibiting the ubiquitin-proteasome pathway. **a** Cells co-transfected with pEGFP-C1-WFDC3 and pDsRed-Express-C1-ERβ were observed under a confocal microscope. Colocalization was shown by merge (yellow). Nuclei were stained by Hoechest 33342 (blue). **b** and **c** WFDC3 interacts with ERβ in the transfected cells. Cells were transiently transfected with the indicated plasmids, and co-immunoprecipitations were performed using an anti-Flag antibody (**b**) or anti-Myc antibody (**c**). **d** and **e** His-ERβ directly interacted with GST-WFDC3 but not GST alone by in vitro GST pull-down assay (**d**) and His-tag pull-down assay (**e**), respectively. **f** The CHX chase assays show that WFDC3 increases ERβ stability in CRC cells. **g** Co-treatment with MG132 and CHX partially minimizes the upregulation of ERβ induced by WFDC3 overexpression. **h** WFDC3 decreases poly-ubiquitination of ERβ. 293 T cells were transfected with Myc-ERβ plasmid, HA-Ub plasmid, and Flag-WFDC3 or Flag-vector plasmids. Cell lysates were immunoprecipitated with an anti-Myc antibody or control IgG, followed by immunoblotting using anti-ubiquitin (Ub). Data are expressed as mean ± SD of at least three independent experiments. Statistics were performed using unpaired Student’s *t*-test, ***P < 0.001.

We next attempted to determine the potential protein-protein interaction between ERβ and WFDC3 by using a laser scanning confocal microscopy. The colocalization of WFDC3 and ERβ was observed in the cytoplasm of LoVo cells (Fig. 4a). The Co-IP results also confirmed that Myc-ERβ was found in Flag-WFDC3 immunoprecipitates (Fig. 4b), while Flag-WFDC3 was detected in Myc-ERβ immune complexes through reciprocal immunoprecipitation (Fig. 4c). Therefore, these findings demonstrate that WFDC3 can interact with ERβ. However, Co-IP revealed no physical interaction between WFDC3 and ERα (Fig. S6a, b). Moreover, to determine whether there was a direct interaction between WFDC3 and ERβ in vitro, GST/His-tag pull-down assays were performed using bacterially expressed GST/His-fused proteins. As shown in Fig. 4d, His-tagged ERβ was pulled down by GST-WFDC3 but not by GST alone. Consistently, GST-WFDC3, but not GST alone, bound to His-tagged ERβ (Fig. 4e), indicating direct binding between WFDC3 and ERβ in vitro.

Previous studies have suggested that ERβ can be degraded via the ubiquitin-proteasome pathway [5]. More interestingly, we found that ERβ protein stability was regulated by WFDC3. After inhibition of protein synthesis by cycloheximide (CHX), WFDC3 overexpression significantly increased ERβ protein stability (Fig. 4f). Moreover, in the presence of the proteasome inhibitor MG132 after CHX pretreatment, the stabilization effect of WFDC3 on ERβ did not further increase the ERβ protein level (Fig. 4g). These data suggested that WFDC3 increased ERβ levels by inhibiting its proteasome-dependent degradation.

ERβ is known to be degraded by ubiquitination [5], thus a ubiquitination-based IP assay was performed to determine the influence of WFDC3 on ERβ ubiquitination. The results show that WFDC3 could reduce ERβ ubiquitination levels in 293 T cells (Fig. 4h), suggesting that WFDC3 increases ERβ stability by interfering with the ubiquitin-proteasome protein degradation pathway.

### WFDC3 represses TGFBR1 expression through ERβ-mediated transcription

**Fig. 5:**
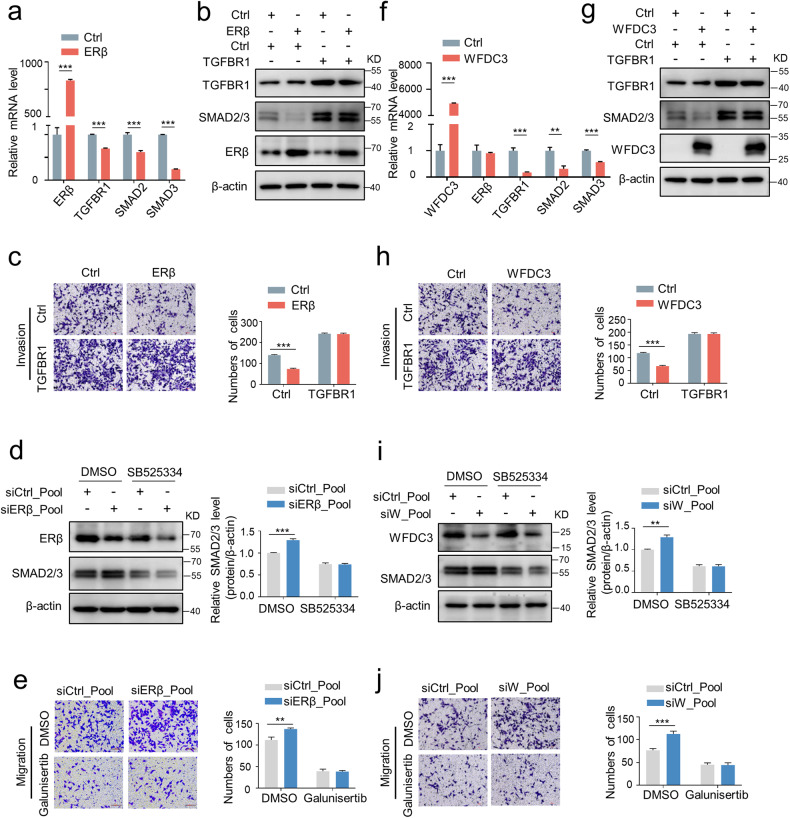
ERβ and WFDC3 attenuates CRC cell invasion through the suppression of TGFBR1. **a** ERβ overexpression inhibits mRNA expression of TGFBR1, SMAD2, and SMAD3. **b** TGFBR1 overexpression rescues the ERβ-mediated reduction in SMAD2/3 protein expression levels. **c** Matrigel invasion assays of CRC cells co-transfected with Flag-TGFBR1 and Myc-ERβ or control plasmid. **d** Effect of SB525334 on ERβ depletion-induced SMAD2/3 downregulation in LoVo cells. **e** Effect of galunisertib on ERβ depletion-induced cell migration in LoVo cells. **f** WFDC3 overexpression inhibits mRNA expression of TGFBR1, SMAD2, and SMAD3. **g** TGFBR1 overexpression rescued the WFDC3-mediated decrease in SMAD2/3 protein expression levels. **h** Matrigel invasion assays of CRC cells co-transfected with Flag-TGFBR1 and Flag-WFDC3 plasmids. The bar graph indicates the number of invaded cells. **i** Effect of SB525334 on WFDC3 depletion-induced SMAD2/3 downregulation in LoVo cells. **j** Effect of galunisertib on WFDC3 knockdown-induced cell migration in LoVo cells. Bar graphs indicate the relative protein levels compared with β-actin or the number of migrated cells. Data are expressed as mean ± SD of at least three independent experiments. Statistics were performed using unpaired Student’s *t*-test, **P < 0.01, ***P < 0.001.

Accumulating evidence also indicates the link between the ERβ and TGFβ pathways in cancer progression [15–17]. This led us to speculate that WFDC3 could be involved in the ERβ/TGFβ signaling pathway. As a transcription factor, ERβ controls the TGFβ pathway by regulating TGFBR1 expression in osteoblasts [17]. We first confirmed the regulation of TGFBR1 by ERβ in CRC cells, and the results indicated that overexpression of ERβ could downregulate TGFBR1 and the downstream factors SMAD2/3 at both the mRNA and protein level (Fig. 5a, b). Moreover, TGFBR1 overexpression rescued the ERβ-induced reduction of SMAD2/3 levels, as well as cell migration and invasion in LoVo cells (Fig. 5b, c and Fig. S7a, b). In the presence of the TGFBR1 inhibitor SB525334, TGFBR1 failed to increase the migration of CRC cells (Fig. S7c), and ERβ knockdown did not upregulate SMAD2/3 levels (Fig. 5d). Similarly, with the treatment of galunisertib, a small molecule inhibitor of TGFβ signaling pathway, ERβ knockdown could not increase the migration of CRC cells (Fig. 5e), indicating that TGFBR1 is a downstream gene of ERβ.

Considering the observed ERβ-induced inhibition of CRC migration through TGFBR1 and the role of WFDC3 in ERβ protein stability, we sought to explore the impact of TGFBR1 on WFDC3-mediated inhibition of migration. The results revealed that overexpression of WFDC3 could inhibit both mRNA and protein expression levels of TGFBR1 and SMAD2/3 (Fig. 5f, g and Fig. S7d). We then ectopically expressed TGFBR1 in WFCD3-overexpressing cells. The rescue of TGFBR1 expression significantly increased the SMAD2/3 levels (Fig. 5g and Fig. S7d) and cell migration (Fig. 5h and Fig. S7e). With the treatment of SB525334, WFDC3 knockdown could not increase SMAD2/3 levels (Fig. 5i). Moreover, blockade of TGFβ pathway by galunisertib impaired the WFDC3 depletion-induced increase of the migration in CRC cells (Fig. 5j), suggesting that TGFβ signaling is downstream of WFDC3.

**Fig. 6:**
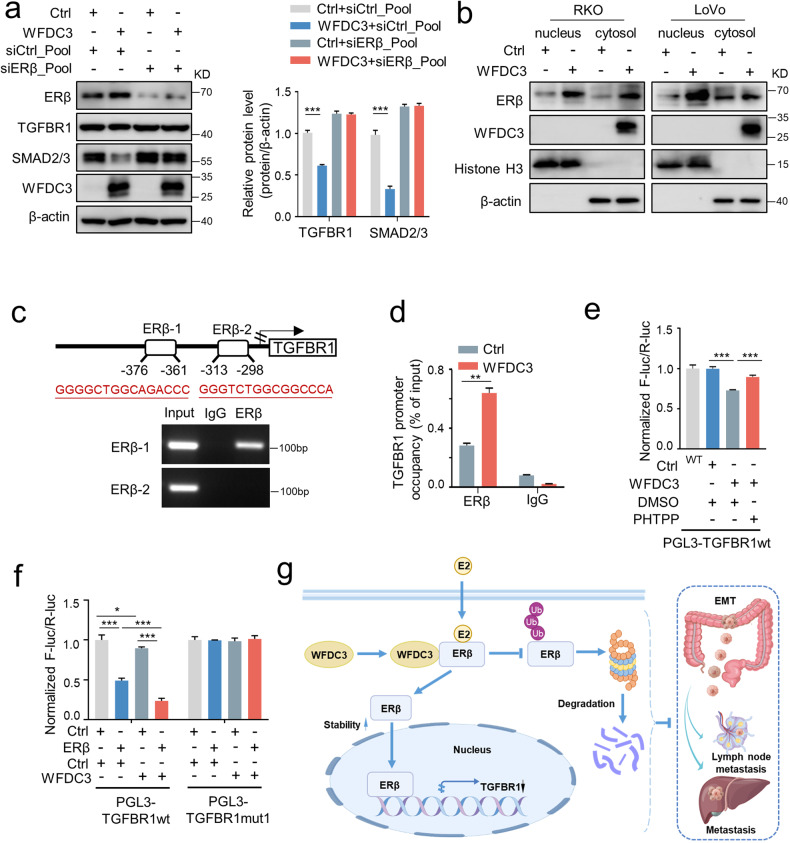
WFDC3 promotes ERβ-mediated repression of TGFBR1 transcription. **a** ERβ depletion rescues the WFDC3-mediated reduction of TGFBR1 and SMAD2/3 at the protein levels. Bar graphs indicate the relative protein levels compared with β-actin. **b** Western blot analysis of ERβ in nucleus and cytoplasmic fractions of RKO and LoVo cells transfected with WFDC3 or control. Histone H3 and β-actin were used as nucleus and cytoplasmic markers, respectively. **c** Two potential ERβ-binding sites were predicted on the TGFBR1 promoter region. ChIP assay was performed in LoVo. **d** ChIP assay-coupled to qPCR to analyze the effects of WFDC3 on the binding of ERβ to the promoter of TGFBR1. **e** The activity of the TGFBR1 promoter was determined by luciferase reporter assays in LoVo cells with WFDC3 overexpression and PHTPP treatment. **f** Luciferase reporter assay indicated the effects of ERβ and WFDC3 on TGFBR1 promoter (wild type or mutated) in LoVo cells with or without ERβ or WFDC3 overexpression. Firefly luciferase activity was measured and normalized to Renilla luciferase activity. Data are expressed as mean ± SD of at least three independent experiments. Statistics were performed using unpaired Student’s *t*-test, *P < 0.05, **P < 0.01, ***P < 0.001. **g** A schematic model illustrates the potential mechanisms by which WFDC3 facilitates estrogen-induced inhibition in metastasis through the ERβ/TGFBR1 axis. Created with Figdraw.com.

To further clarify the regulatory relationships among ERβ, WFDC3, and TGFBR1, we knocked down ERβ in WFDC3-overexpressing LoVo cells. Interestingly, knocking down ERβ retarded the decreased expression of TGFBR1 and SMAD2/3 induced by WFDC3 overexpression (Fig. 6a), indicating that ERβ is required for the WFDC3-mediated regulation of TGFBR1. Together, these data suggest that WFDC3 and ERβ work coordinately to ensure that TGFBR1 functions in a concerted manner.

Because the nuclear translocation of ERβ is critical for its transcriptional role, we performed nuclear and cytoplasmic fractionation assays to determine whether the subcellular distribution of ERβ was regulated by WFDC3. Our results showed that WFDC3 significantly increased the accumulation of ERβ in the nucleus (Fig. 6b).

Furthermore, to elucidate how WFDC3 and ERβ suppress TGFBR1 transcription, chromatin immunoprecipitation (ChIP) assays were performed to determine the binding of ERβ to the promoter of TGFBR1. We predicted two putative sites (ERβ-1 and ERβ-2) that are located within the TGFBR1 promoter region and revealed that ERβ could bind to the ERβ-1 site located at −376 to −361 bp from the transcriptional start site of TGFBR1 (Fig. 6c). To further verify these results, we also constructed two promotor-mutant TGFBR1 constructs (TGFBR1 mut1 and TGFBR1 mut2) for dual-luciferase activity assays. As shown in Fig. S7f, overexpressing ERβ significantly reduced transcriptional activities from the wild type (WT) TGFBR1 promoter and TGFBR1-mut2. However, ERβ had no obvious effect on the transcriptional efficiency of TGFBR1-mut1. Thus, ERβ modulated TGFBR1 transcription primarily through the site where mut1, but not mut2, was generated.

More importantly, ChIP-qPCR analysis confirmed that WFDC3 overexpression resulted in a significant increase of ERβ binding to the TGFBR1 promoter (Fig. 6d). Inhibiting ERβ with PHTPP partially blocked the WFDC3-induced inhibition of TGFBR1 promoter activity (Fig. 6e). Moreover, simultaneously overexpressing WFDC3 and ERβ further reduced the transcriptional activity of the WT TGFBR1 promoter, but not TGFBR1-mut1 (Fig. 6f). These results suggest that WFDC3 can suppress the transcription of TGFBR1 in an ERβ-dependent manner (Fig. 6g).

### WFDC3 is downregulated in CRC and correlated with favorable prognosis

**Fig. 7:**
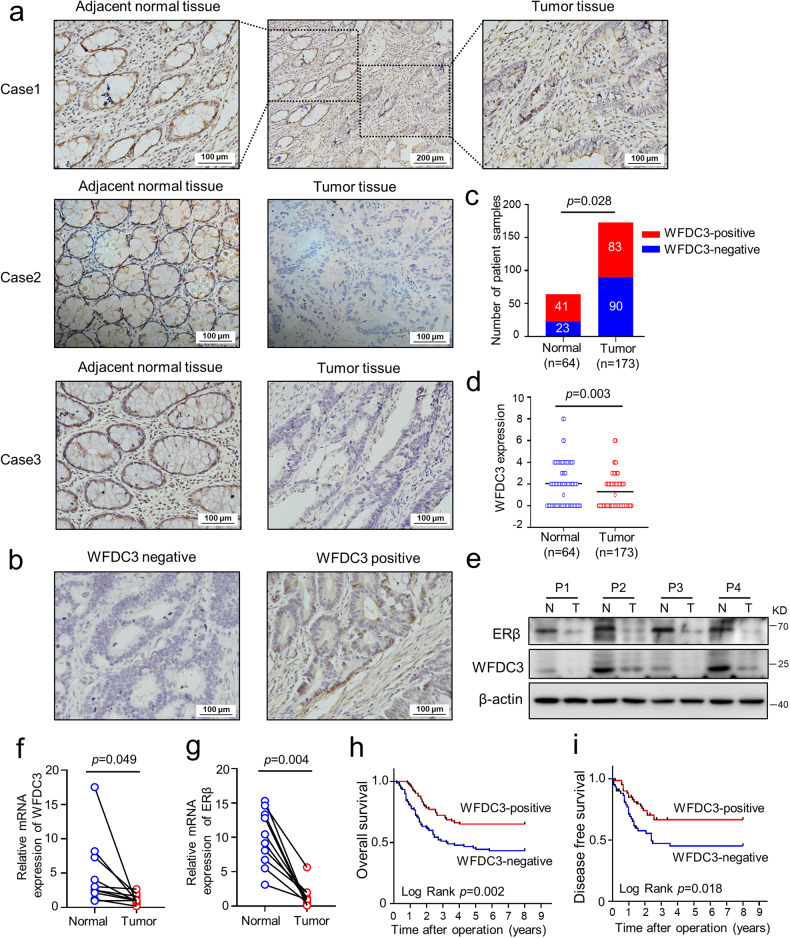
WFDC3 expression in CRC tissues. **a** Representative images of WFDC3 immunohistochemistry (IHC) staining in CRC tissues and adjacent normal tissues. **b** Representative images of negative and positive expression of WFDC3 in CRC tissues (Magnification, 200×, Scale bars, 100 μm). **c** The number of WFDC3-positive and WFDC3-negative expression in adjacent normal tissues and CRC tissues. Statistical significance was determined by Pearson *χ*^2^‐test. **d** Quantification of WFDC3 expression according to IHC scores in normal and tumor tissues. IHC scores were compared by unpaired Student’s *t*-test. **e** WFDC3 protein expression in four paired CRC tissues and adjacent normal tissues was assessed by western blot. Western blot image has been cropped for presentation. **f** and **g** Relative mRNA expression of WFDC3 (**f**) and ERβ (**g**) in representative paired CRC tissues and adjacent normal tissues (blue: normal; red: tumor). Statistics were performed using paired Student’s *t*-test. **h** Kaplan–Meier analysis of overall survival in CRC patients categorized according to WFDC3 status. **i** Kaplan–Meier analysis of disease-free survival in CRC patients categorized according to WFDC3 status.

Finally, to determine the clinical significance and prognostic role of WFDC3 in CRC patients, we examined WFDC3 expression in 173 human CRC tissues via IHC staining (Fig. 7a, b). The results of the IHC assay showed that positive WFDC3 expression was detected in 41out of 64 (64.1%) adjacent normal tissues and in only 83 out of 173 (48.0%) CRC tissues (Fig. 7c). Similarly, according to our IHC scoring standard, WFDC3 protein expression were significantly downregulated in tumor tissues compared with adjacent normal tissues (Fig. 7d, *P* = 0.003). In addition, we found that lower WFDC3 levels were significantly associated with lymph node metastasis (*P* = 0.007), distant metastasis (*P* = 0.005), and TNM stage (*P* = 0.015, Table 1).

**Table 1:** Correlations between WFDC3 expression and clinicopathological characteristics in CRC patients.

Variables	Cases	WFDC3 expression		
		Positive	Negative	*P*-value
Age (years)				0.984
≤60	102	49	53	
>60	71	34	37	
Gender				0.867
Male	72	34	38	
Female	101	49	52	
Tumor location				0.123
Colon	98	42	56	
Rectum	75	41	34	
Tumor size (cm)				0.513
≤4	104	52	52	
>4	69	31	38	
Depths of invasion				0.252
T1/T2	20	12	8	
T3/T4	153	71	82	
Lymph node metastasis				**0.007**
Negative	48	31	17	
Positive	125	52	73	
Distance metastasis				**0.005**
Negative	104	59	45	
Positive	69	24	45	
TNM stage				**0.015**
I/II	96	54	42	
III/ IV	77	29	48	
Differentiation				0.073
Well	2	1	1	
Moderate	126	67	59	
Poor	45	15	30	

Statistical significance was determined by Pearson *χ*^2^‐test, *P*-values in bold were statistically significant.

To further validate our results, four representative paired CRC and adjacent normal tissue samples were subjected to western blot and qRT-PCR analysis. The WFDC3 protein levels were decreased in CRC tissues compared to adjacent normal tissues. A similar trend was observed for ERβ expression levels (Fig. 7e). The qRT-PCR results also confirmed that WFDC3 and ERβ levels in CRC samples were dramatically lower than those in adjacent normal tissues (Fig. 7f, g).

By further validating the clinical significance of WFDC3 in our cohort of 173 CRC patients, we confirmed WFCD3 was significantly associated with a favorable prognosis in patients. Kaplan–Meier analysis of overall survival (OS) and disease-free survival (DFS) revealed that patients with high WFDC3 expression levels had favorable OS (*P* = 0.002) and DFS (*P* = 0.018, Fig. 7h, i). Furthermore, we evaluated the prognostic value of WFDC3 in the CRC patients by univariate and multivariate Cox regression analyses. In the univariate analysis, we found that WFDC3 expression was a significant predictor of OS (hazard ratio [HR]: 0.570; 95% confidence interval [CI]: 0.382–0.851; *P* = 0.006; Table 1). Moreover, age (*P* = 0.034), gender (*P* = 0.044), depth of invasion (*P* = 0.023), lymph node metastasis (*P* = 0.015), distant metastasis (*P* < 0.001), and TNM stage (*P* < 0.001) were significant variables in the univariate analysis (Table 2). Then, the features with statistical significance in the univariate analysis were chosen to adjust covariates in the multivariate Cox regression analysis. The multivariate survival analysis also indicated that WFDC3 expression is an independent prognostic factor for CRC patient OS (*P* = 0.025, Table 2).

**Table 2:** Univariate and multivariate analysis of overall survival in CRC patients.

Variables		Univariate			Multivariate	
	HR	95%CI	*P*-value	HR	95%CI	*P*-value
Age ( > 60 yr. vs ≤60 yr.)	1.609	1.038-2.496	**0.034**	1.413	0.907-2.202	0.126
Gender (Female vs. Male)	0.636	0.410-0.987	**0.044**	0.583	0.372-0.915	**0.019**
Tumor location (Rectum vs. Colon)	0.635	0.402-1.002	0.051			
Tumor size (>4 vs. ≤4 cm)	1.142	0.731-1.782	0.560			
Depth of invasion (T3/T4 vs. T1/T2)	3.214	1.176-8.789	**0.023**	2.156	0.758-6.136	0.150
Lymph node metastasis (N1/N2 vs.N0)	1.980	1.144-3.427	**0.015**	1.468	0.828-2.602	0.189
Distance metastasis(M1 vs.M0)	2.992	1.919-4.605	**<0.001**	2.600	1.648-4.103	**<0.001**
TNM stage (III/IV vs. I/II)	2.545	1.628-3.979	**<0.001**			
Tumor differentiation (Poor vs. Well/moderate)	1.218	0.745-1.989	0.432			
WFDC3 expression (Positive vs. Negative)	0.570	0.382–0.851	**0.006**	0.644	0.438-0.946	**0.025**

*HR* hazard ratio, *CI* confidence interval. *P*-values, calculated by Cox proportional hazard regression analysis, which in bold were statistically significant.

## Discussion

The estrogen receptor ERβ is the predominantly expressed estrogen receptor in the colon and has been demonstrated to protect against CRC [31]. Therefore, understanding the underlying mechanisms of ERβ signaling may lead to new therapeutic approaches for CRC. In this study, we identified that WFDC3 plays a critical role in the inhibitory effects of estrogen on CRC metastasis by stabilizing ERβ protein and targeting its downstream gene, TGFBR1 (Fig. 6g). Moreover, increased WFDC3 expression in CRC patients may indicate a better prognosis. These findings point to WFDC3 as a potential biomarker for estrogen/ERβ pathway-targeted therapies in CRC patients.

WFDC3, also known as WAP14, encodes a member of the WFDC-domain family. While spliced transcript variants of WFDC3 have been identified, their full-length nature has not been well determined. Previous studies have found that WFDC3 is involved in lipopolysaccharide (LPS)-induced inflammation in mice and could be a target to adjuvant therapies for epididymitis [24]. Additionally, WFDC3 is associated with risk for homogenous antinuclear antibody pattern (ANA^H^) in combination with homozygosity for dog leukocyte antigen (DLA), which leads to the occurrence of Systemic Lupus Erythematosus-related diseases [25]. Moreover, a previous study demonstrated that WFDC3 is one of the most downregulated genes in the ventral prostate of ERβ-/- (ERβ knockout) mice [26]. However, the role of WFDC3 in cancer has not been elucidated.

In this study, we defined, for the first time, WFDC3 as a tumor suppressor that prevents EMT and tumor metastasis in CRC, which is consistent with the function of WFDC1 and WFDC2 in prostate cancer [23, 32]. However, members of the WFDC family such as WFDC2 and WFDC4 promote tumor progression in ovarian cancer and CRC [20, 22, 33, 34], respectively, indicating the complicated roles of different WFDC subfamily members. Their functions as oncogenes or tumor suppressors are dependent on the specific tissue and tumor type. Additionally, we found that high levels of WFDC3 in tumor tissues predict favorable prognosis for CRC patients. Collectively, these data suggest that WFDC3 acts as a prognostic factor for CRC patients.

Estrogen is a crucial sex hormone that plays multiple biological functions including energy homeostasis, bone remodeling, neuroprotection, and is involved in many cancer-related processes [35, 36]. More importantly, estrogens play prominent roles in the development and progression of breast cancer [28], ovarian cancer [29], and lung cancer [30]. Studies of estrogen and estrogen receptors have led to progress in the definitive standard of endocrine therapies for breast cancer using tamoxifen [37]. However, it has been well established that estrogen can either promote or suppress tumor growth according to the cancer type [38]. In contrast to the tumor-promoting role of estrogen in breast cancer [28], accumulating evidence suggests that estrogen has anti-cancer activity in CRC [39–41]. Young women with CRC have improved overall survival compared with men of the same age. However, the protection of hormone is lost when women achieve menopause [40, 41]. In postmenopausal women, hormone replacement therapy with estrogen only could reduce the CRC risk and CRC-specific mortality rate [42]. Despite this, hormone therapy has not been widely used for routine clinical purposes in CRC.

Estrogen has also been shown to regulate the tumor immune microenvironment and suppress CRC growth via the GPER, p38α, and MAPK signaling pathways [27, 43, 44]. Our findings identified, for the first time, the collaborative effects of WFDC3 and estrogen in the inhibition of CRC metastasis. These data raise the possibility of using WFDC3 as a biomarker for therapeutic strategies that target the estrogen/ERβ pathway to ease the burden of CRC.

In principle, the effects of estrogen are mediated through ERs. ERα is a well-known target of endocrine therapy for breast cancer [5]. In contrast, ERβ is the most abundant ER expressed in colorectal tissues and serves as a target for CRC prevention. Due to its important roles, ERβ is associated with a favorable prognosis in CRC patients [6, 31, 45]. However, the application of ERβ as a target in hormone therapy for CRC is not well studied. In this study, we found that overexpression of ERβ impede CRC cell migration, which is consistent with the previous studies [37, 46]. Given that in ERβ-/- mice ventral prostates, WFDC3 is downregulated [26], we explored the association between WFDC3 and the estrogen/ERβ pathway in CRC progression. The results showed that the ERβ antagonist, PHTPP could abolish WFDC3-induced inhibition of CRC migration and invasion, suggesting that ERβ is required for the WFDC3-mediated inhibition of metastasis. These data revealed for the first time that WFDC3 might serve as a valuable target for CRC therapy that targets the ERβ pathway.

It has been reported that ERβ protein is degraded through the ubiquitin-proteasome pathway [5, 47, 48]. Thus, modulation of ERβ expression and stability is a promising potential strategy for cancer therapeutics. NDRG2 promotes ERβ protein stability by inhibiting ubiquitin protein ligase E3A to suppress CRC [48]. Sanchez et al. demonstrated that Mdm2 could interact with ERβ and promote the ubiquitination and degradation of ERβ [47]. In this study, we demonstrated that WFDC3 could upregulate ERβ expression mainly at the protein level, and the interaction between WFDC3 and ERβ inhibits the ubiquitination and degradation of ERβ.

The TGFβ/SMAD pathway promotes tumor invasion and metastasis by inducing EMT. Although previous studies have demonstrated mutual regulation between the ERβ and TGFβ pathways in cancer progression [17, 49, 50], this is the first study to suggest that WFDC3 might be the upstream regulator of ERβ-mediated TGFβ/SMAD signaling.

As a transcription factor, ERβ can regulate the transcription of ERβ-targeted genes [9, 51]. ERβ directly downregulates VEGF-A transcription through the estrogen response element (ERE), as well as indirectly represses HIF-1-mediated transcription [51]. Consistent with a previous study [17], we found that ERβ could inhibit TGFBR1 transcriptional activity. Additionally, we identified a new potential ERβ-binding site in the TGFBR1 promoter, which is distinct from those previously reported [17], indicating the regulatory mechanisms of ERβ for controlling TGFBR1 transcriptional activity. Moreover, WFDC3 promoted nuclear transfer of ERβ and enhanced the effects of ERβ on reducing the transcriptional activity of the TGFBR1 promoter. It raises the possibility that WFCD3 recruits ERβ more efficiently for the ERβ/TGFBR1 regulatory axis. In addition, TGFBR1 could rescue the ERβ or WFDC3-induced reduction of CRC cell migration, suggesting that TGFBR1 is indeed a downstream effector of WFDC3 and ERβ, and repurposing the TGFβ pathway inhibitor galunisertib, which is being investigated in clinical trials, could be a strategy for the treatment of CRC patients with low levels of WFDC3 or ERβ expression.

Although our observations reveal the clinical significance of WFDC3 in CRC patients, larger cohorts of CRC patients with detailed clinicopathologic parameters are needed to validate these findings. Moreover, considering the functional role of WFDC3 in ERβ/TGFBR1-mediated regulation of metastasis, we speculate that WFDC3 might be a therapeutic indicator. Further studies are required to verify the potential of ERβ-targeted therapies to treat CRC patients with high WFDC3 expression.

Overall, this study identified WFDC3 as a tumor suppressor that inhibits CRC metastasis via ERβ-dependent TGFβ signaling. The role of WFDC3 in the ERβ/TGFBR1 axis suggests that WFDC3 could be a promising target for potential ERβ-specific therapies in CRC patients.

## Supplementary information

Supplementary Figures

Supplementary Methods and Tabled

checklist

Original western blots

## Data Availability

All data generated or analyzed during this study are included in this article and its additional files.
