# Supplementary material for: WFDC3 inhibits tumor metastasis by promoting the ERβ-mediated transcriptional repression of TGFBR1 in colorectal cancer
Source: Cell Death Dis. 2023 Jul 13;14(7):425. doi: 10.1038/s41419-023-05956-0 (PMC10345115; doi:10.1038/s41419-023-05956-0)
Supplement: Supplementary file 1 — Supplementary Figures [file 41419_2023_5956_MOESM1_ESM.pdf]

Fig S1

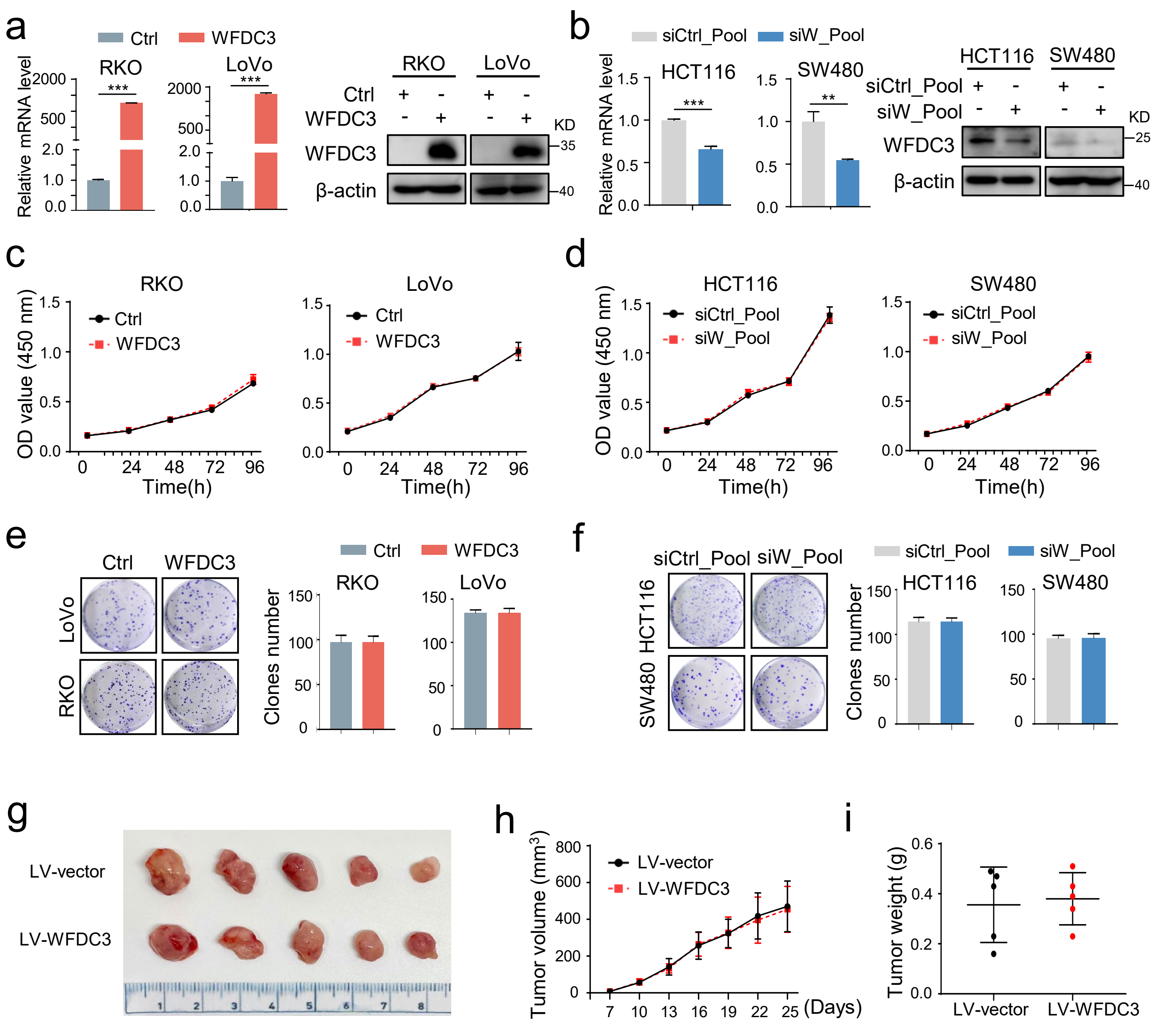

Figure S1. **WFDC3 had no significant effects on CRC cells proliferation. a** WFDC3 overexpression in RKO and LoVo cells was measured using qRT-PCR and western blot. **b** WFDC3 knockdown with siRNA pool in HCT116 and SW480 cells was assessed using qRT-PCR and western blot. **c** and **d** Overexpression **(c)** or knockdown **(d)** of WFDC3 had no significant effect on CRC cell proliferation. **e** and **f** Overexpression **(e)** or knockdown **(f)** of WFDC3 had no obvious effect on the colony formation of CRC cells. The bar graph shows the number of cells. Data are expressed as mean±SD of at least three independent experiments. Statistics were performed using unpaired Student's *t*-test, ***P < 0.001. g-i Tumors (n=5) extracted from mice implanted with RKO-Ctrl or RKO-WFDC3 cells **(g)**. Tumor masses were harvested from the corresponding xenografts on day 25, tumor volumes **(h)** and tumor weights **(i)** were measured on the indicated days.

Fig S2

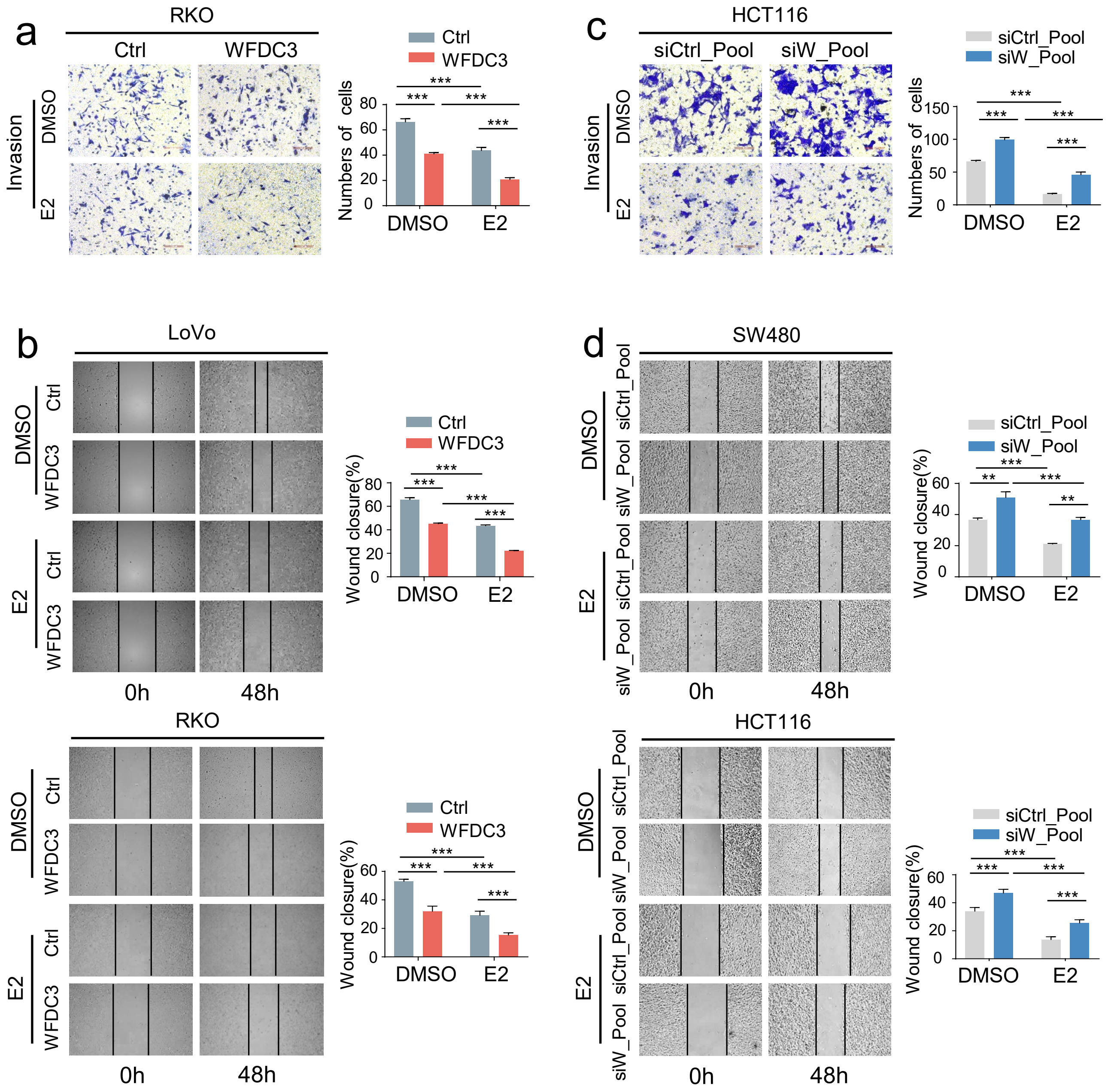

Figure S2. **WFDC3 promotes the inhibitory effects of estrogen in migration of CRC cells. a** Matrigel invasion assay of WFDC3-overexpressing RKO cells treated with estrogen or DMSO control. **b** Wound healing assays of WFDC3-overexpressing LoVo cells and RKO cells treated with estrogen or DMSO. **c** Matrigel invasion assay of HCT116 cells transfected with pooled WFDC3 siRNA and treated with estrogen or the DMSO control. **d** Wound healing assays of WFDC3-depleted SW480 or HCT116 cells treated with estrogen or the DMSO control. The bar graph indicates the number of migrated cells and percentage of wound closures. Data are expressed as mean ± SD. Statistics were performed using unpaired Student's *t*-test, $^{**}P < 0.01$, $^{***}P < 0.001$.

Fig S3

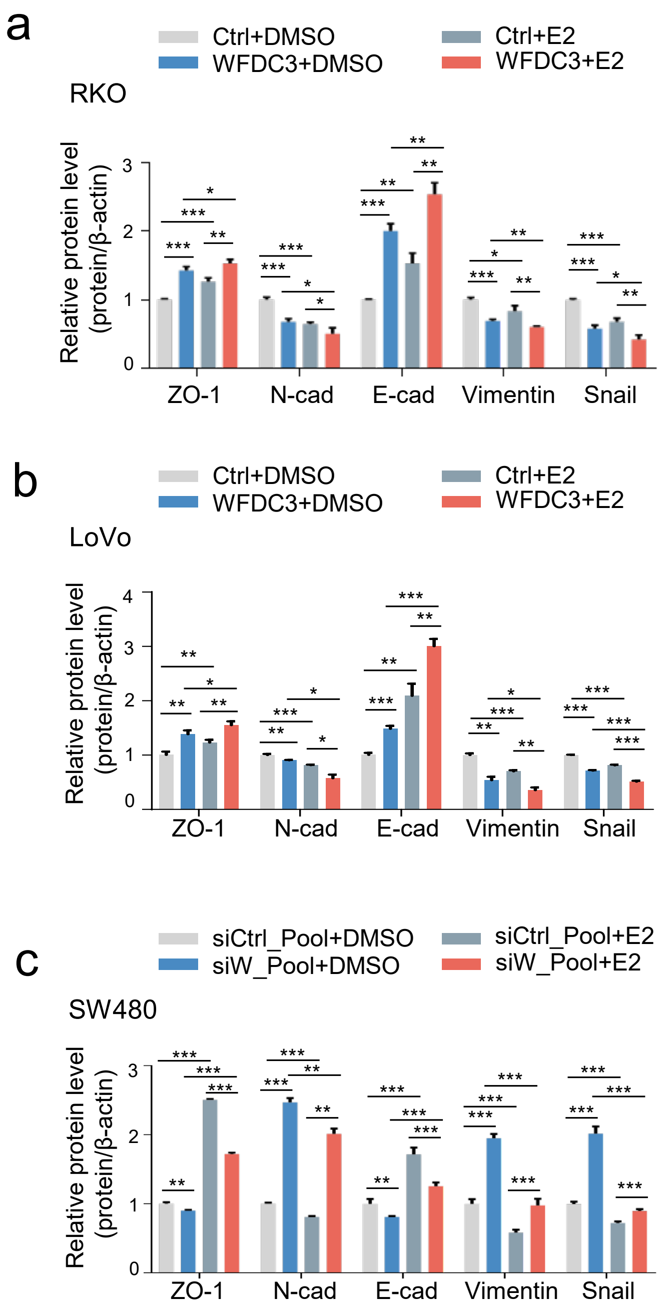

Figure S3. Quantification of changes in epithelial-mesenchymal transition (EMT)-related protein levels in Figure 1. a-c Quantification of relative protein levels normalized to β-actin in Figure 1c-e. Data are expressed as mean±SD of at least three independent experiments. Statistics were performed using unpaired Student's *t*-test, **P* < 0.05, ***P* < 0.01, ****P* < 0.001.

Fig S4

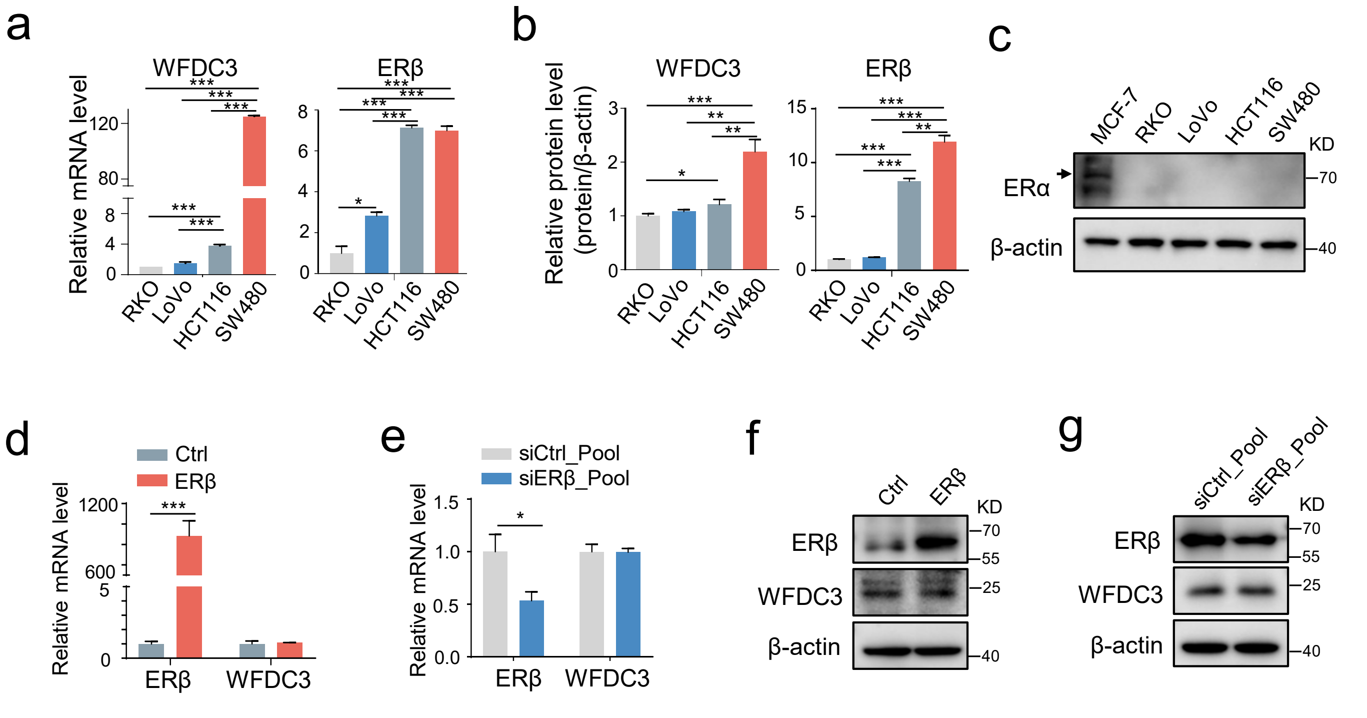

Figure S4. ERβ has no significant effect on WFDC3 expression. a The mRNA expression levels of WFDC3 and ERβ in CRC cell lines. **b** Quantification of relative protein levels normalized to β-actin in Figure 3c. Bar graphs indicate the relative protein levels compared with β-actin. **c** The protein expression levels of ERα in five cell lines. **d** and **e** Treatment with ERβ plasmid **(d)** or pooled ERβ siRNA **(e)** exerts no significant effect on the WFDC3 mRNA expression levels. **f** and **g** ERβ overexpression **(f)** or depletion **(g)** exerts no significant effect on WFDC3 protein expression levels. Western blot images have been cropped for presentation. Data are expressed as mean ± SD of at least three independent experiments. Statistics were performed using unpaired Student's *t*-test, **P* < 0.05, ****P* < 0.001.

Fig S5

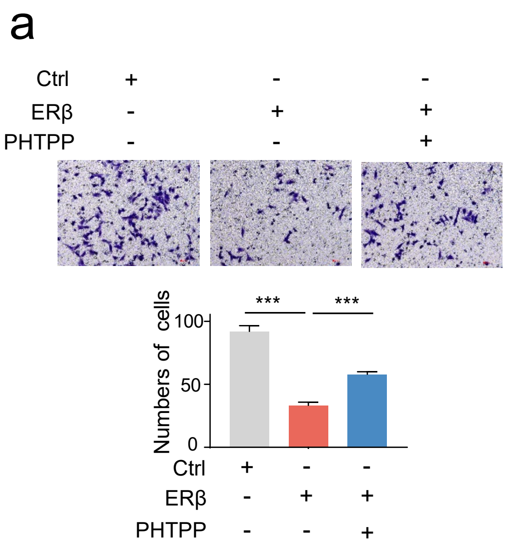

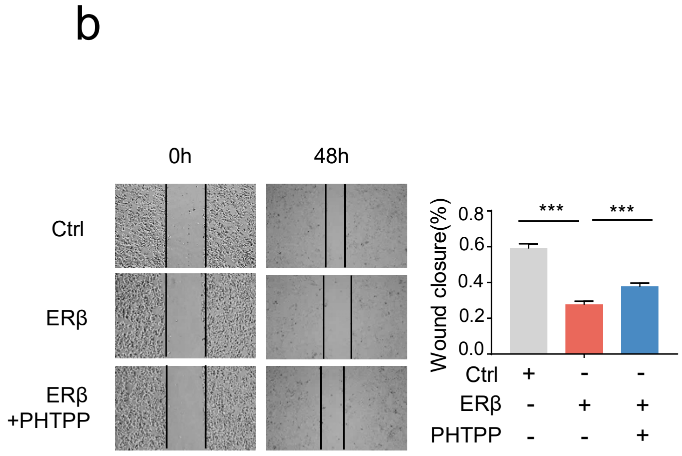

Figure S5. ERβ inhibits CRC cell migration. a and **b** Transwell migration **(a)** and wound healing assays **(b)** was performed in LoVo cells transfected with Myc-ERβ in the presence of PHTPP or DMSO control. Data are expressed as mean±SD of at least three independent experiments. Statistics were performed using unpaired Student's *t*-test, ***$P < 0.001$.

Fig S6

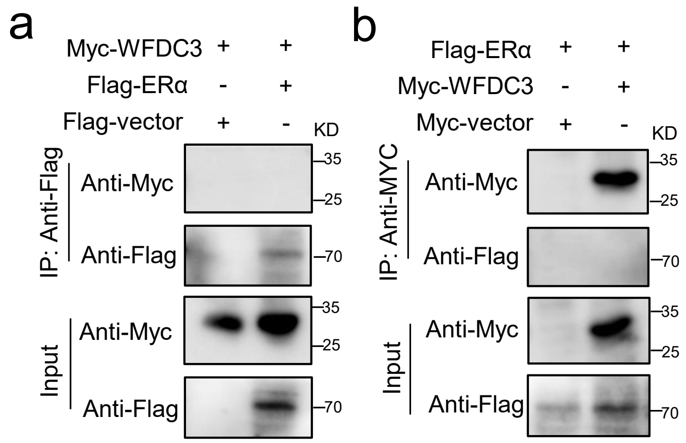

Figure S6. WFDC3 has no interaction with ERα. a and **b** WFDC3 had no interaction with ERα in transfected cells. Cells were transiently transfected with the indicated plasmids, and co-immunoprecipitations were performed using an anti-Flag antibody **(a)** or anti-Myc antibody **(b)**.

Fig S7

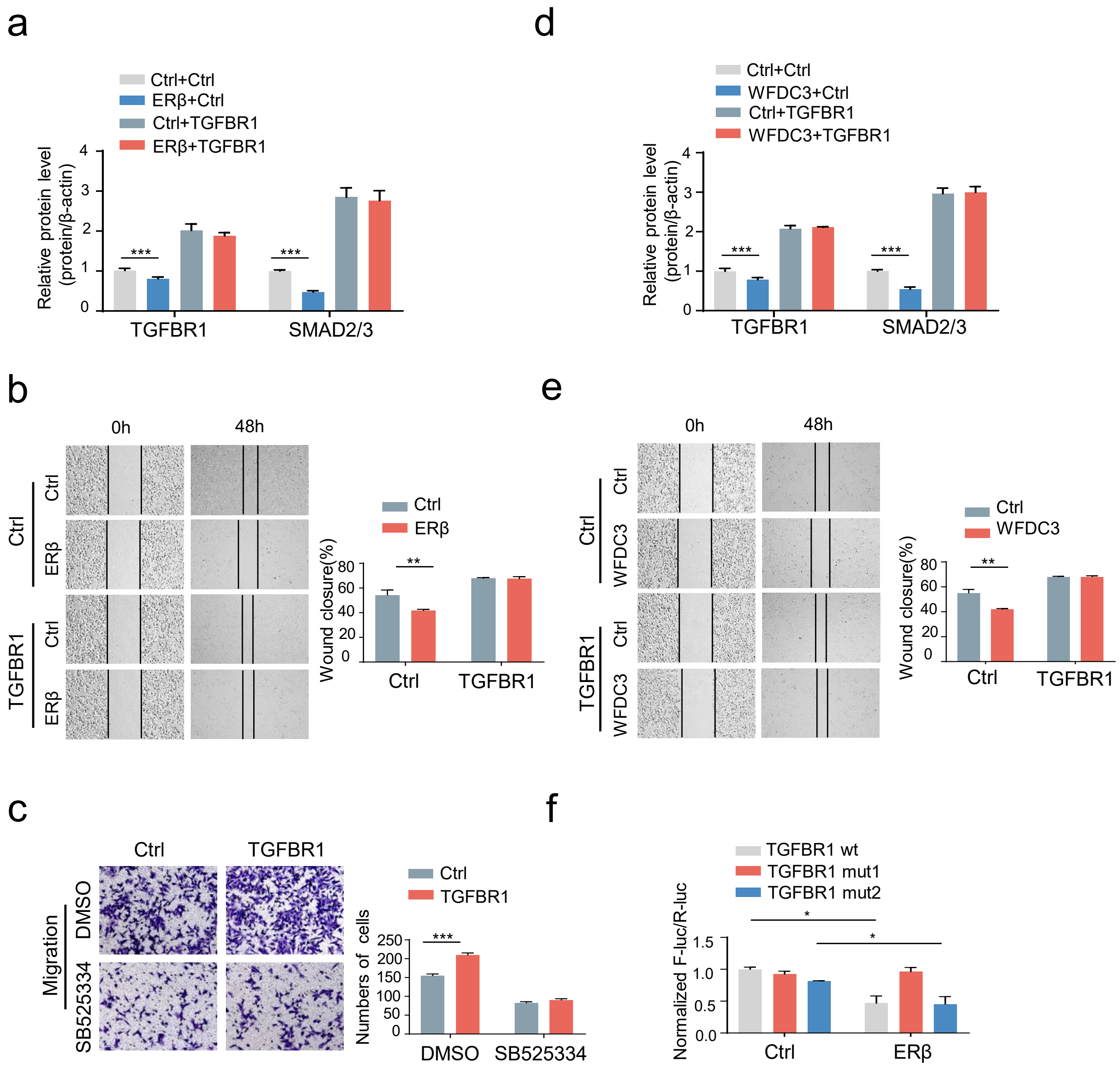

Figure S7. WFDC3 inhibits CRC cell invasion by enhancing ERβ-induced repression in TGFBR1 transcription. a Quantification of relative protein levels normalized to β-actin in Figure 5b. **b** Wound healing assay of LoVo cells co-transfected with Myc-ERβ and Flag-TGFBR1 plasmids. **c** Transwell migration assays of LoVo cells transfected with Flag-TGFBR1 in the presence of SB525334 treatment or DMSO control. The bar graph indicates the number of migrated cells. **d** Quantification of relative protein levels normalized to β-actin in Figure 5g. **e** Wound healing assay of LoVo cells co-transfected with Flag-WFDC3 and Flag-TGFBR1 plasmids. The bar graph indicates the percentage of wound closures. **f** Putative ERβ binding sites in TGFBR1 promoter were identified by luciferase reporter assay. Firefly luciferase activity was measured and normalized to Renilla luciferase activity. Data are expressed as mean ± SD of at least three independent experiments. Statistics were performed using unpaired Student's *t*-test, *$P < 0.05$, **$P < 0.01$, ***$P < 0.001$.
