# Supplementary material for: WFDC3 inhibits tumor metastasis by promoting the ERβ-mediated transcriptional repression of TGFBR1 in colorectal cancer
Source: Cell Death Dis. 2023 Jul 13;14(7):425. doi: 10.1038/s41419-023-05956-0 (PMC10345115; doi:10.1038/s41419-023-05956-0)
Supplement: Supplementary file 2 — Supplementary Methods and Tabled [file 41419_2023_5956_MOESM2_ESM.docx]

**WFDC3 inhibits tumor metastasis by promoting the ERβ-mediated transcriptional repression of TGFBR1 in colorectal cancer**

Tianqi Liu^1,#^, Min Zhao^2,#^, Lin Peng^1^, Jiangbo Chen^1^, Pu Xing^1^, Pin Gao^1^, Lei Chen^1^, Xiaowen Qiao^1^, Zaozao Wang^1^, Jiabo Di^1^, Hong Qu^3,*^, Beihai Jiang^1,*^, Xiangqian Su^1,*^

^#^These authors contributed equally to this work.

*Corresponding authors.

**Supplementary Materials and Methods**

**Cell lines, cell culture and reagents**

Human CRC cell lines (RKO, LoVo, HCT116, and SW480), human breast cancer cell line MCF7, and human embryonic kidney cell line HEK293T were obtained from American Type Culture Collection (ATCC, Manassas, VA, USA) and cultured in Dulbecco’s Modified Eagle Medium (DMEM; HyClone, China) supplemented with 10% fetal bovine serum (FBS; Gibco, USA) and penicillin/streptomycin. Cell lines were maintained in a humidified atmosphere containing 5% CO_2_ at 37°C as described. Testing for mycoplasma contamination is carried out every six months and STR profiles of the above cells are tested.

For estrogen-related studies, CRC cell lines were maintained in serum-free DMEM without phenol red (HyClone) for 12 hours prior to treatment with 17β-estradiol (E2) (E8875, Sigma-Aldrich, USA) and ERβ antagonist PHTPP (S8686, Selleck, USA).

To block the TGFBR1 pathway, 10 μM Galunisertib (Selleck, USA) or 10 μM SB525334 (Selleck, USA) was added with DMSO as vehicle control.

**Plasmid, small interfering RNA (siRNA), and lentivirus transfections**

For overexpression, the full-length WFDC3 cDNA was cloned into the pCMV3-C-FLAG plasmid and Ubi-MCS-SV40-firefly_Luciferase-IRES-PuroMycin lentiviral vector (Shanghai Genechem Co., Ltd., China). The full-length ERβ cDNA sequence was cloned into the pCMV-Myc plasmid and the full-length TGFBR1 cDNA sequence was cloned into the pcDNA3 expression vector. For knockdown, short hairpin RNA (shRNA) of human WFDC3 was cloned into the hU6-MCS-Ubiquitin-firefly_Luciferase-IRES-puromycin lentiviral vector (Shanghai Genechem Co., Ltd., China). ON-TARGET plus SMARTpool WFDC3 siRNA (Cat #L-013334-01-0005), ON-TARGET plus SMARTpool ERβ siRNA (Cat #L-003402-00-0005), and control siRNA were purchased from Dharmacon. The SMARTpool reagent contains a mixture of four individual siRNAs targeting the same gene. Cells were seeded and transiently transfected with indicated plasmids or siRNAs using Lipofectamine 2000 (Invitrogen, USA) following the manufacturer’s suggestions. For lentiviral transfection, cells were infected with the lentiviral vectors and were then selected using 2 μg/mL puromycin.

**Immunofluorescence staining and confocal microscopy**

To investigate the effect of WFDC3 expression on EMT biomarkers, CRC cells were grown on 12 mm coverslips and then incubated with or without 17-beta-estradiol (E2) for 24 hours after transfection of WFDC3. Cells were fixed with 4% PFA, permeabilized with 0.2% Triton-X-100, blocked with 10% goat serum in PBS, and then incubated with the primary antibodies of E-cadherin (1:400, 3195, CST) or N-cadherin (1:200, 13116S, CST) at 4°C overnight. The cells were then stained with Hoechst 33342 (10 μg/mL) and images were taken with a Leica SP5 confocal system.

To observe the colocalization of WFDC3 and ERβ, human full-length WFDC3 and ERβ cDNA were cloned into the pEGFP-C1 and pDsRed-Express-C1 vector, respectively. LoVo cells were co-transfected with pEGFP-C1-WFDC3 and pDsRed-Express-C1-ERβ. After 48 hours, the cells were incubated with Hoechst 33342 (10 μg/mL) for 15 minutes and observed under a Leica SP5 confocal system.

**Quantitative real-time PCR (qRT-PCR) and** **chromatin immunoprecipitation (ChIP) assays**

Total RNA was extracted from CRC cells using Trizol (Invitrogen) according to the manufacturer’s instructions. Reverse transcription was performed with the Reverse Transcription Kit (Promega, Madison, WI, USA). qRT-PCR was performed as previously described (1). GAPDH was used as an internal reference gene. The chromatin immunoprecipitation (ChIP) assay was carried out using the SimpleChIP® Plus Sonication Chromatin IP Kit (CST, #9002). The primer sequences used in this study were listed in Table S1.

**Western blot analysis**

Western blot procedures were followed as described in a previous study (1). The primary antibodies and dilutions used were listed in Table S2. For nucleo-cytoplasmic fractionation, the nuclear and cytoplasmic fractions were extracted using Nuclear and Cytoplasmic Protein Extraction Kit (Beyotime, P0028, China) according to the manufacturer’s protocol. β-actin and Histone H3 was used to normalize samples. The protein densities were quantified using ImageJ software (NIH, USA). Original western blots for the results are supplied as Supplementary Materials.

**Cell proliferation and colony formation assays**

CRC cell growth was determined by CCK8 assays following manufacturer’s instructions (CK04, Dojindo, Japan). The absorbance values were measured at 450 nm with a microplate reader (Bio-Rad, Hercules, CA, USA).

To assess colony formation ability, CRC cells (800 cells/well) were seeded into a 6-well plate and incubated for nearly two weeks. Cell colonies were fixed with paraformaldehyde (PFA), stained with 0.1% crystal violet, and then counted.

**Transwell assays**

Transwell chambers (Costar, USA) were used for migration and invasion assays. For the invasion assay, the upper chamber was pre-coated with Matrigel. Cells in serum-free medium were seeded into the upper chamber, with the lower chamber containing 800 μL of culture medium supplemented with 15% FBS. After incubation, migrated cells were fixed with 4% PFA and stained with crystal violet. Images were photographed and migrated cells counted in three randomly selected fields under a microscope.

**Wound healing assays**

CRC cells were transfected and cultured to reach 90% confluence in 6-well plates, then wounded gaps were created using a 10 µL pipette tip. The cells were then washed with PBS and cultured in serum-free medium. The gaps were photographed at 0 hours and 48 hours after the wound was created using a microscope (Leica Microsystems, Wetzlar, Germany) and the wound widths were measured using ImageJ software.

**GST pull-down and his-tag pull-down assays**

The coding regions of WFDC3 and ERβ were inserted into the pGEX-4T-1 and pET-28a (+) vectors, respectively, which were then transformed into the *E. coli* strain BL21 (DE3). Proteins were expressed and purified in accordance with the manufacturer’s instructions (Amersham, Chicago, IL, USA). For GST and His-tag pull-down assays, purified His-ERβ protein was mixed with GST or GST-WFDC3 and incubated with glutathione Sepharose 4B beads (GE Healthcare, Chicago, IL, USA) and Ni-NTA agarose (QIAGEN, Hilden, Germany), respectively, followed by western blot with anti-GST and anti-His tag antibodies (Beijing Zhongshan Golden Bridge Biotechnology Co Ltd., Beijing, China).

**Cycloheximide (CHX) chase assay**

LoVo cells were co-transfected with Myc-ERβ plasmid and Flag-WFDC3 or Flag-vector. After 36 hours, cells were treated with 100 μg/mL CHX for different time points. Cell lysates were then processed for ERβ degradation using western blots.

**Poly-ubiquitination assay**

HEK293T cells were transfected with HA-tagged ubiquitin plasmid, Myc-ERβ, and Flag-WFDC3 or Flag-vector for 48 hours. Then, the cells were treated with 10 μM Z-Leu-Leu-Leu-al (MG132) for 6 hours and harvested, lysed with cell lysis buffer, and immunoprecipitated with c-Myc antibody. The ubiquitinated ERβ proteins were detected by western blot using the anti-ubiquitin antibody (1:200, sc-8017, Santa Cruz Biotechnology, USA).

**References**

1. Zhuang H, Tan M, Liu J, Hu Z, Liu D, Gao J, et al. Human epididymis protein 4 in association with annexin ii promotes invasion and metastasis of ovarian cancer cells. Mol Cancer 2014;13:243.

**Table S1.** Relevant primer sequences.

| Gene | Primer sequences | |
| --- | --- | --- |
| WFDC3 | Forward  Reverse | 5′- CTTGCTCTTGGGTCTCTGGAATC -3′  5′- CTTAGGAATGTCTCGGCAGATCC -3′ |
| GAPDH | Forward  Reverse | 5′-GGACTCATGACCACAGTCCATG -3′  5′- CAGGGATGATGTTCTGGAGAGC -3′ |
| ERβ | Forward  Reverse | 5′- CTTACCTGTAAACAGAGAGACAC- 3′  5′- TTGCGCCGGTTTTTATCGATTGT -3′ |
| TGFBR1 | Forward  Reverse | 5′- ACATGATTCAGCCACAGATACC -3′  5′- GCATAGATGTCAGCACGTTTG -3′ |
| SMAD2 | Forward  Reverse | 5′- CGTCCATCTTGCCATTCACG -3′  5′-CTCAAGCTCATCTAATCGTCCTG -3′ |
| SMAD3 | Forward  Reverse | 5′- TGGACGCAGGTTCTCCAAAC -3′  5′- CCGGCTCGCAGTAGGTAAC -3′ |
| ERβ-1-CHIP | Forward | 5′- ACCCATCGCTCTACCCGGCCCT -3′ |
|  | Reverse | 5′- ATCGGCTCCCGGCTCCGAGAG -3′ |
| ERβ-2-CHIP | Forward | 5′- TCTCGGAGCCGGGAGCCGAT -3′ |
|  | Reverse | 5′- CGAGCGCCGGTTTCTGGCC -3′ |

**Table S2.** Antibodies for western blot.

| Antibody | Manufacture | Item number | Species |
| --- | --- | --- | --- |
| WFDC3 | Proteintech, China | Cat#24917-1-AP | Rabbit |
| ZO-1 | Cell Signaling Technology, USA | Cat#8193S | Rabbit |
| N-Cadherin | Cell Signaling Technology, USA | Cat#13116S | Rabbit |
| E-Cadherin | Cell Signaling Technology, USA | Cat#3195S | Rabbit |
| Vimentin | Cell Signaling Technology, USA | Cat#5741S | Rabbit |
| Snail | Cell Signaling Technology, USA | Cat#3879S | Rabbit |
| ERβ | Proteintech, China | Cat#14007-1-AP | Rabbit |
| ERα | Proteintech, China | Cat#21244-1-AP | Rabbit |
| TGFBR1 | Abcam, USA | Cat#ab235178 | Rabbit |
| SMAD2/3 | Cell Signaling Technology, USA | Cat#8685S | Rabbit |
| Flag | Sigma-Aldrich, USA | Cat# F1804 | Mouse |
| c-Myc | Clontech Laboratories, USA | Cat# 631206 | Mouse |
| β-actin | Sigma-Aldrich, USA | Cat#A1978 | Mouse |
| His | ZSGB-BIO, China | Cat#TA-02 | Mouse |
| GST | ZSGB-BIO, China | Cat#TA-03 | Mouse |
| Histone H3 | Cell Signaling Technology, USA | Cat#4499S | Rabbit |
| goat anti-mouse IgG | ZSGB-BIO, China | Cat#ZB-2305 | Goat |
| goat anti-rabbit IgG | ZSGB-BIO, China | Cat#ZB-2301 | Goat |
