# Supplementary material for: WFDC3 inhibits tumor metastasis by promoting the ERβ-mediated transcriptional repression of TGFBR1 in colorectal cancer
Source: Cell Death Dis. 2023 Jul 13;14(7):425. doi: 10.1038/s41419-023-05956-0 (PMC10345115; doi:10.1038/s41419-023-05956-0)
Supplement: Supplementary file 4 — Original western blots [file 41419_2023_5956_MOESM4_ESM.pdf]

Fig 1

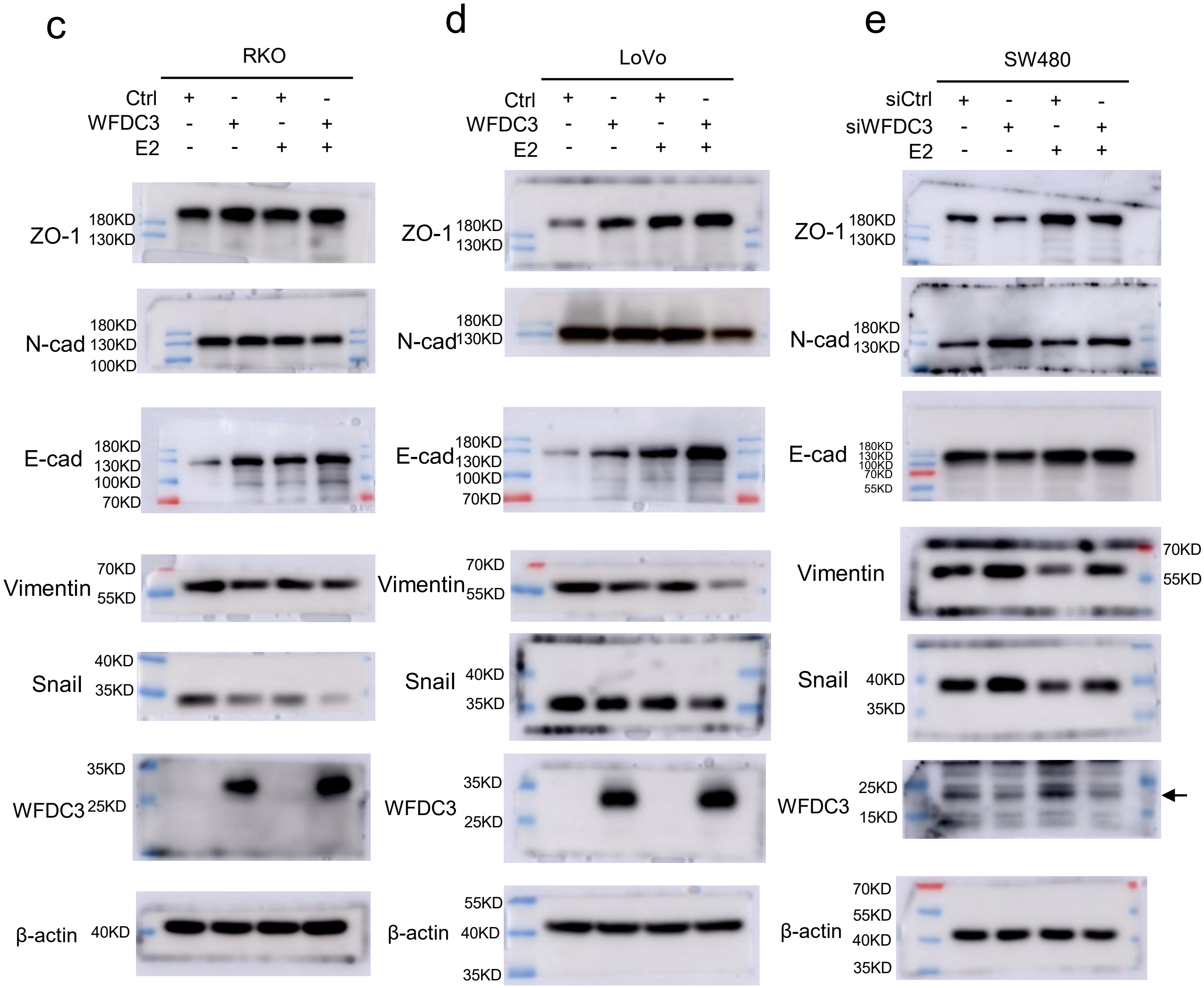

Uncropped blots in Fig.1c-e.

Fig 2

a

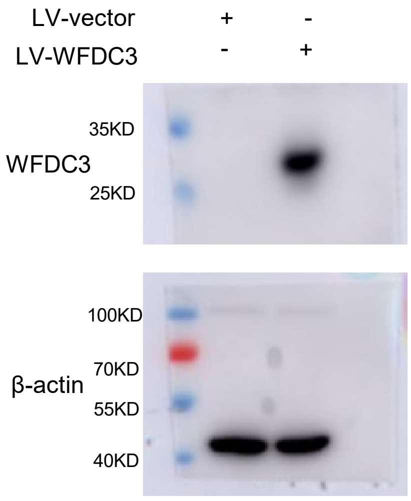

g

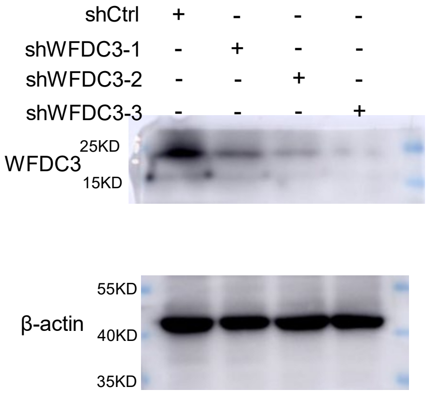

Uncropped blots in Fig.2a, g.

Fig 3

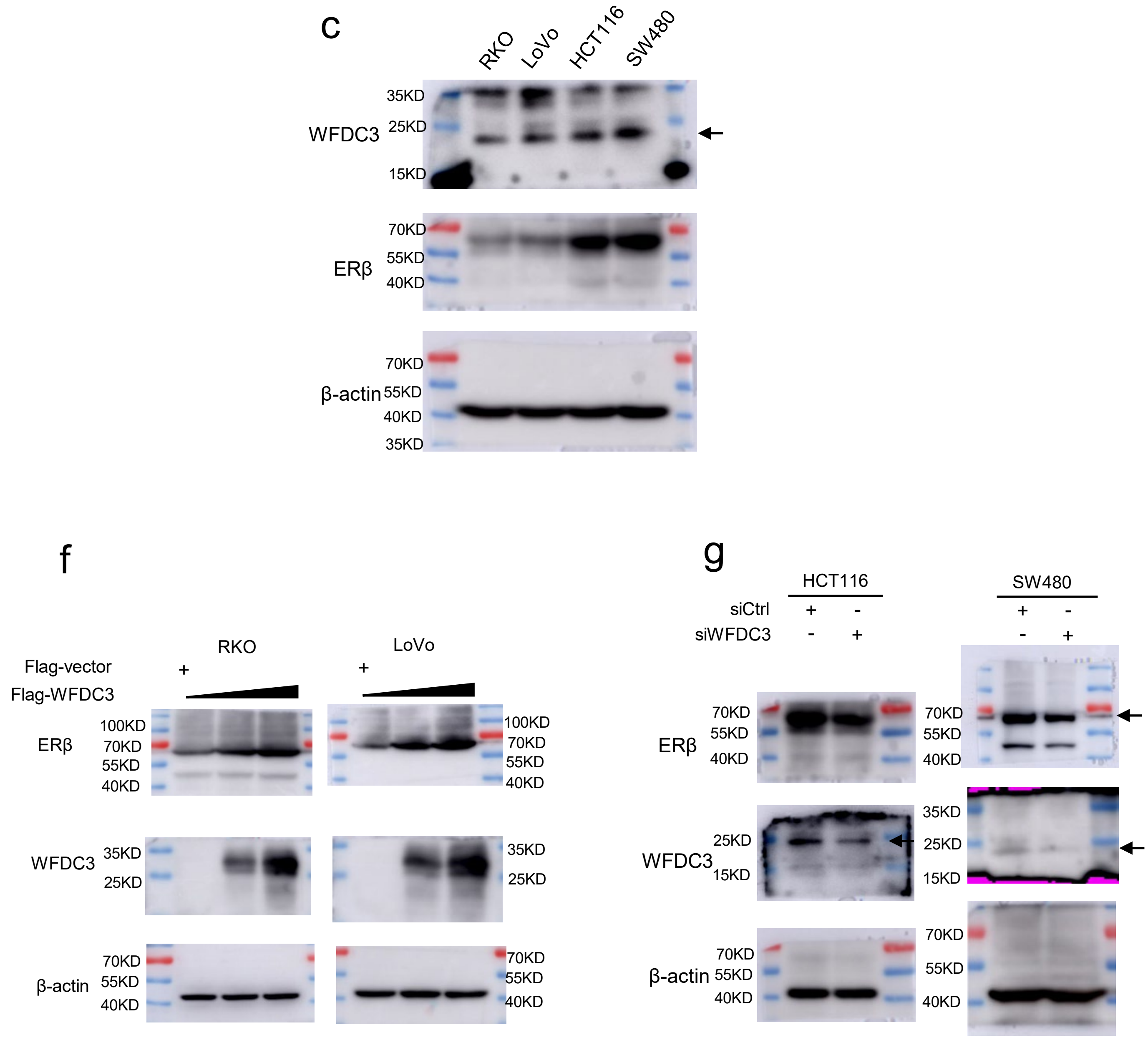

Uncropped blots in Fig.3c,f,g.

Fig 4

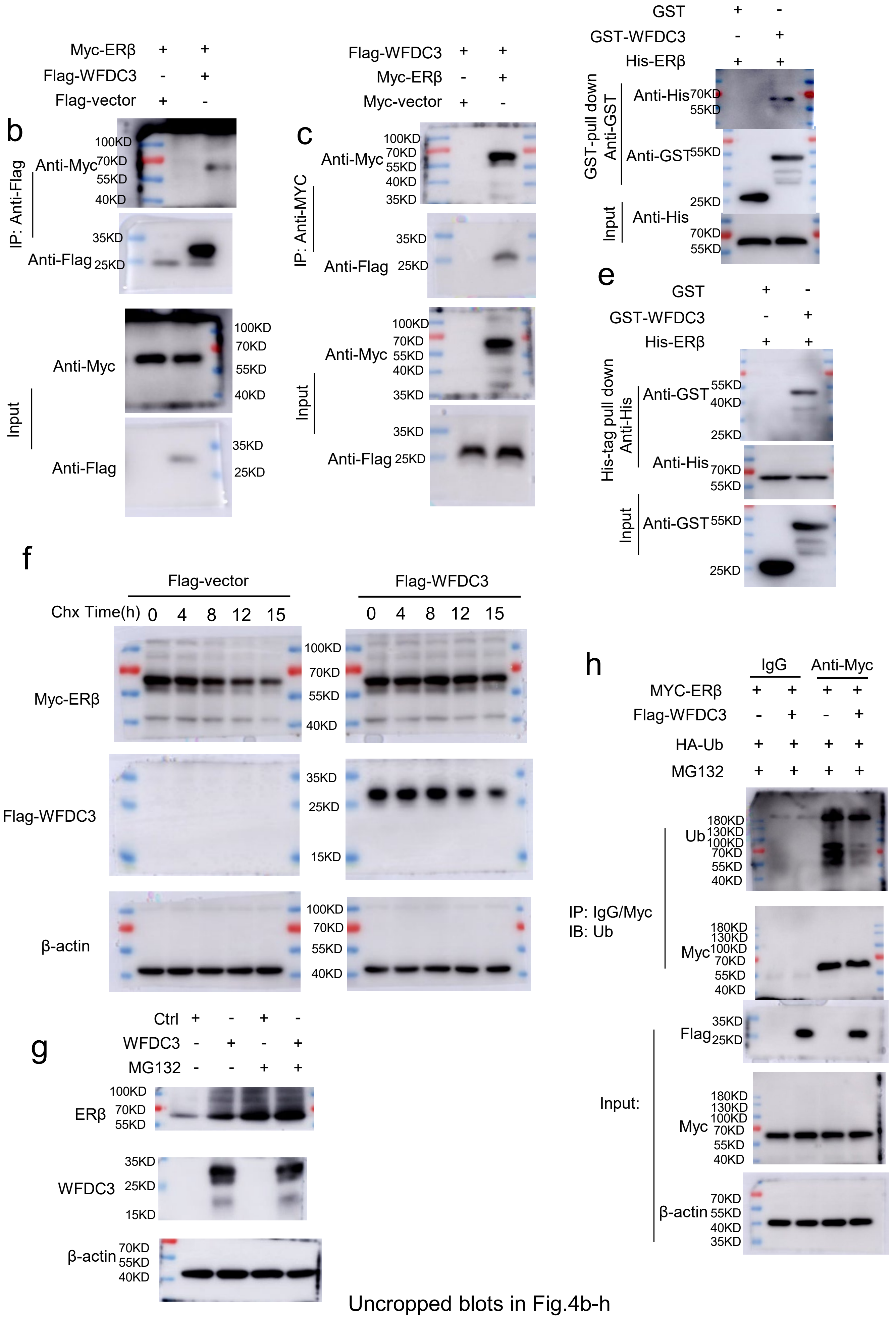

Uncropped blots in Fig.4b-h

Fig 5

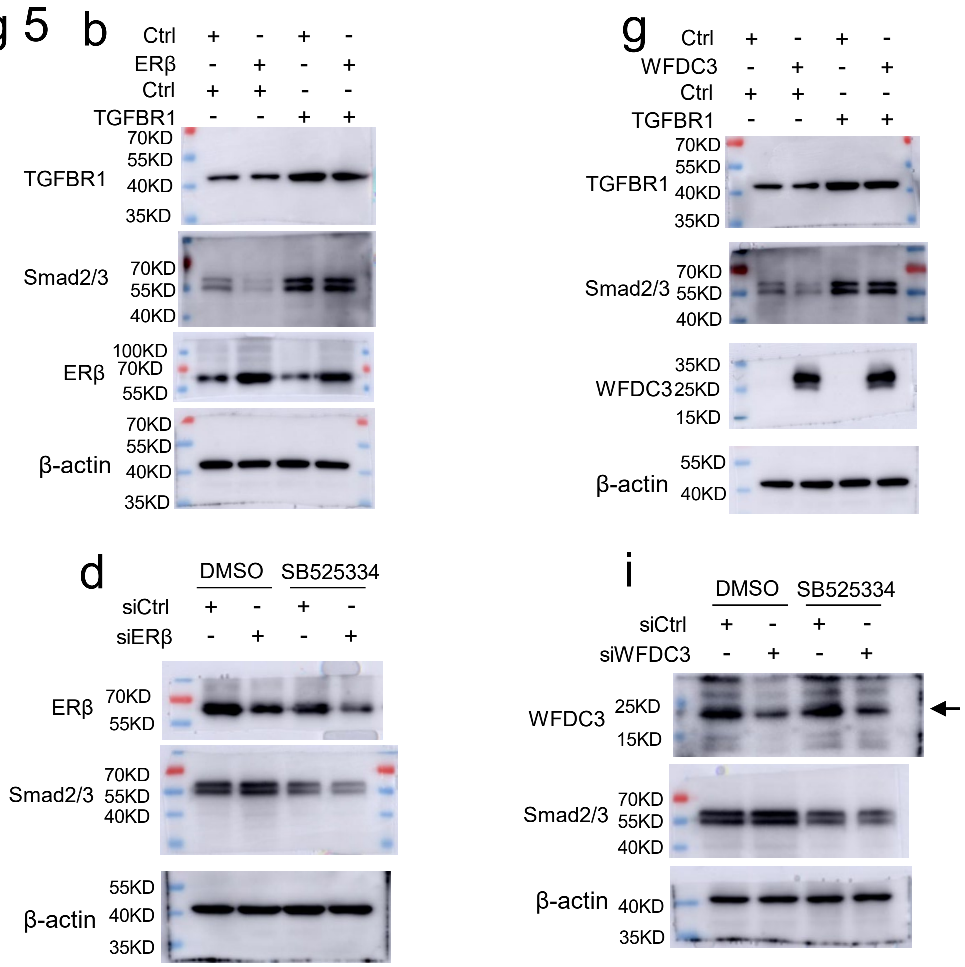

Uncropped blots in Fig.5

Fig 6

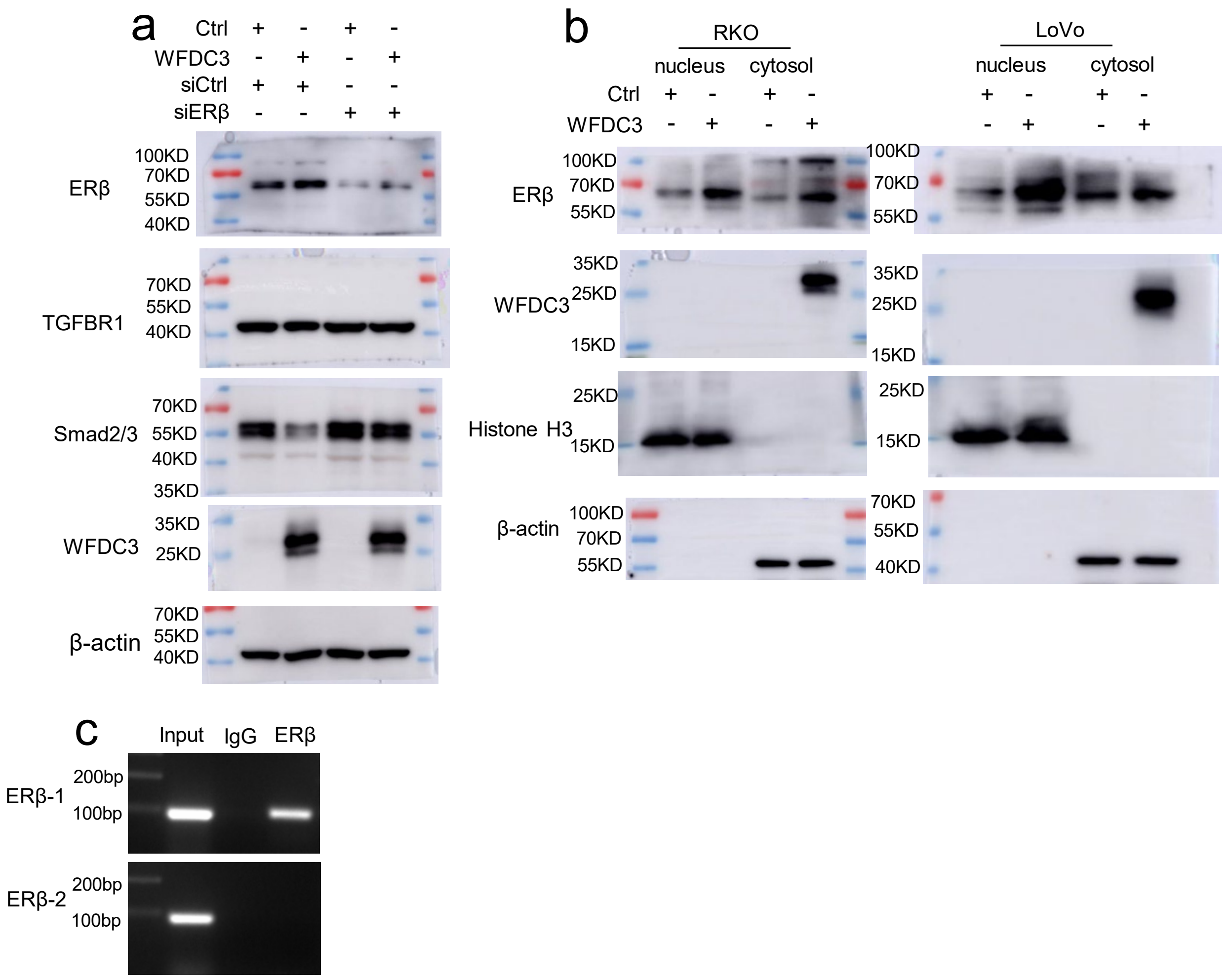

Uncropped blots in Fig.6

Fig 7

f

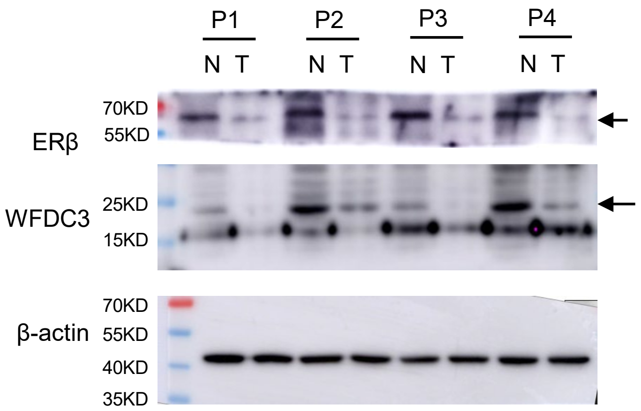

Uncropped blots in Fig.7e .

Fig S1

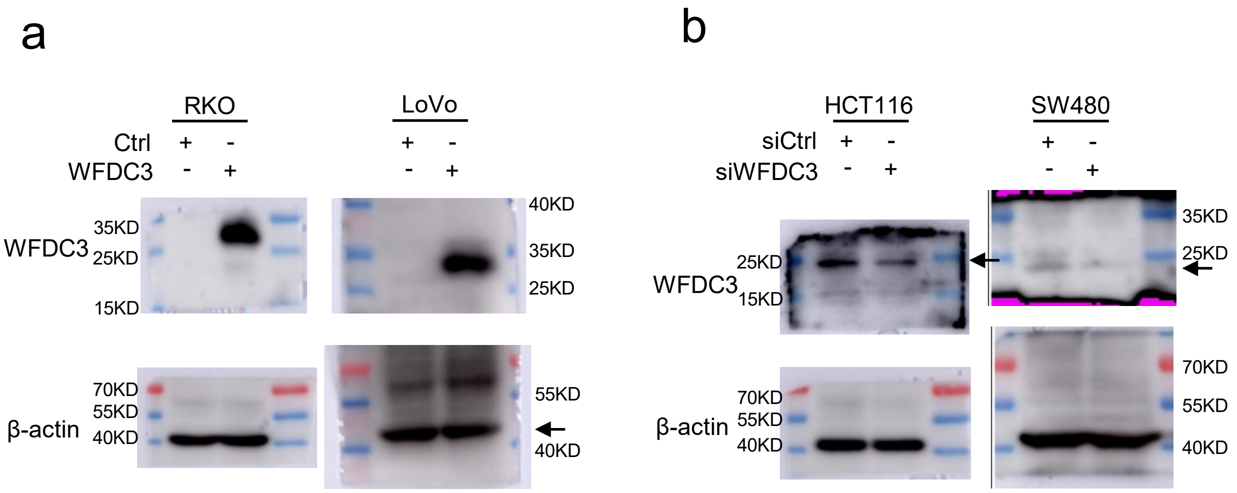

Uncropped blots in Fig.S1a,b

Fig S4

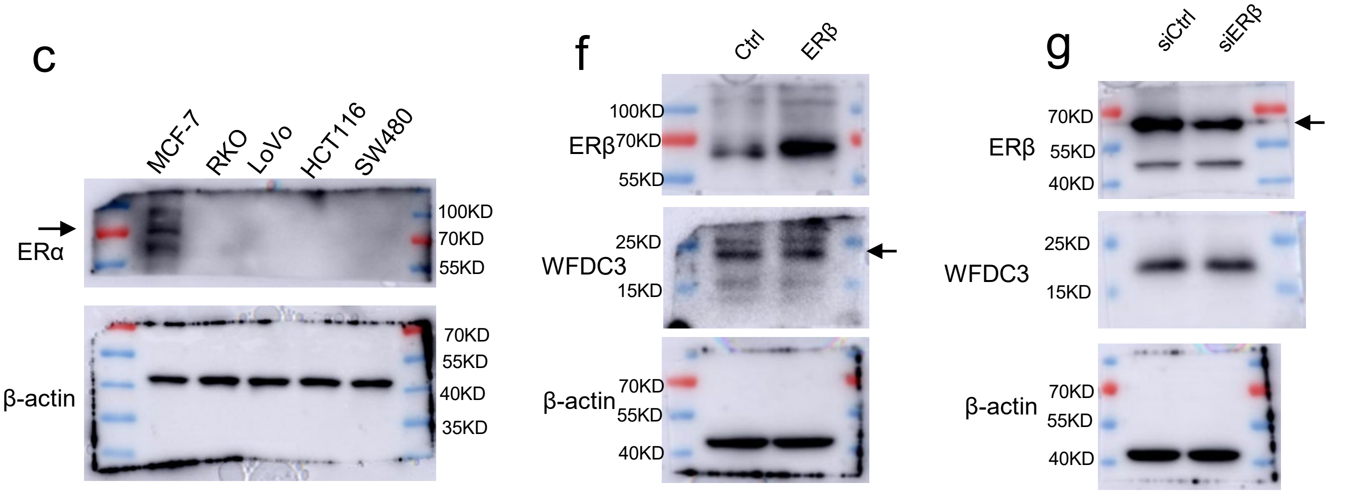

Uncropped blots in Fig.S4c,f,g

Fig S6

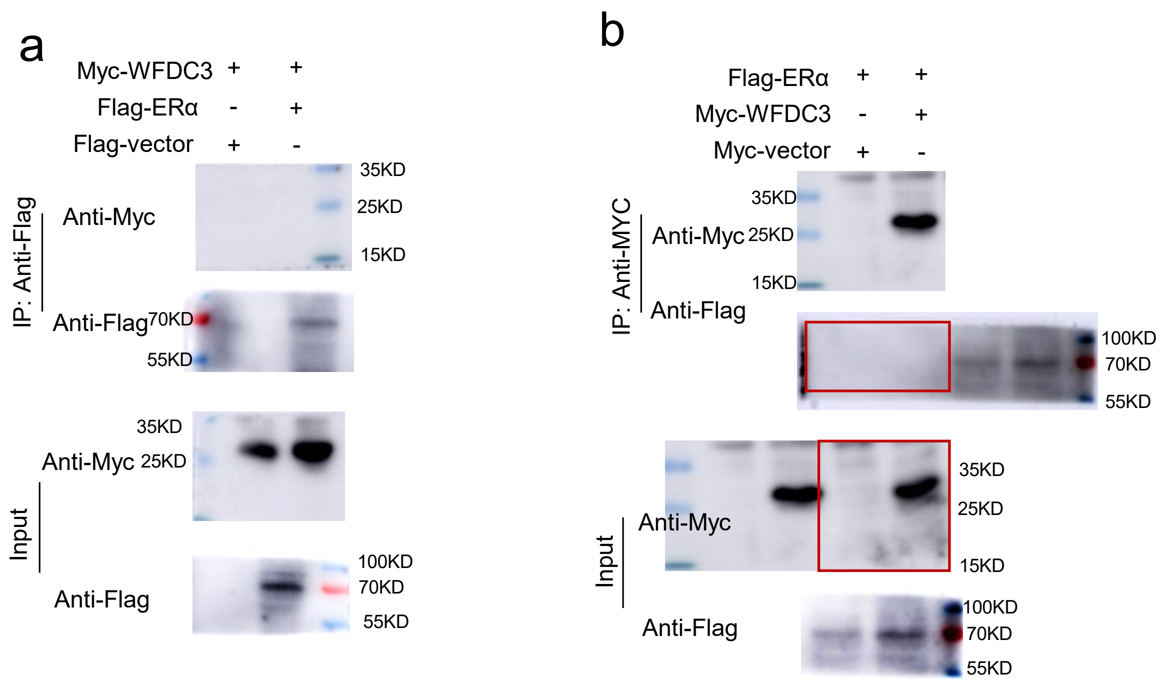

Uncropped blots in Fig.S6
